# LncGMDS-AS1 promotes the tumorigenesis of colorectal cancer through HuR-STAT3/Wnt axis

**DOI:** 10.1038/s41419-023-05700-8

**Published:** 2023-02-27

**Authors:** Deji Ye, Hanshao Liu, Guojun Zhao, Aijun Chen, Yuhang Jiang, Yiming Hu, Dandan Liu, Ningxia Xie, Weifei Liang, Xi Chen, Haohao Zhang, Cuifeng Li, Jingyao Wang, Donglin Sun, Weifeng Chen, Dan Tan, Qi Wang, Hongru Wang, Dianping Yu, Baojin Wu, Mingliang Wang, Shuzhong Cui, Sanhong Liu, Xiaoren Zhang

**Affiliations:** 1 The Sixth Affiliated Hospital, Affiliated Cancer Hospital/Institute and GMU-GIBH Joint School of Life Sciences of Guangzhou Medical University, Qingyuan People’s Hospital, State Key Laboratory of Respiratory Disease, Qingyuan, 511518 Guangdong China; 2 CAS Key Laboratory of Tissue Microenvironment and Tumor, Shanghai Institute of Nutrition and Health, Shanghai Institutes for Biological Sciences, University of Chinese Academy of Sciences, Chinese Academy of Sciences, Shanghai, 200031 China; 3 General Surgery Department, Ruijin Hospital, Shanghai Jiao Tong University School of Medicine, Shanghai, PR China; 4 Shanghai Frontiers Science Center of TCM Chemical Biology, Institute of Interdisciplinary Integrative Medicine Research, Shanghai University of Traditional Chinese Medicine, Shanghai, 201203 China; 5 Shanghai Institute of Biochemistry and Cell Biology, Center for Excellence in Molecular Cell Science, Chinese Academy of Sciences, Shanghai, 200031 China

**Keywords:** Colorectal cancer, Long non-coding RNAs, RNA decay, Tumour biomarkers, Ubiquitylation

## Abstract

Chronic inflammation promotes the tumorigenesis and cell stemness maintenance of colorectal cancer (CRC). However, the bridge role of long noncoding RNA (lncRNA) in linking chronic inflammation to CRC development and progression needs better understanding. Here, we elucidated a novel function of lncRNA GMDS-AS1 in persistently activated signal transducer and transcription activator 3 (STAT3) and Wnt signaling and CRC tumorigenesis. Interleukin-6 (IL-6) and Wnt3a induced lncRNA GMDS-AS1 expression, which was highly expressed in the CRC tissues and plasma of CRC patients. GMDS-AS1 knockdown impaired the survival, proliferation and stem cell-like phenotype acquisition of CRC cells in vitro and in vivo. We performed RNA sequencing (RNA-seq) and mass spectrometry (MS) to probe target proteins and identify their contributions to the downstream signaling pathways of GMDS-AS1. In CRC cells, GMDS-AS1 physically interacted with the RNA-stabilizing protein HuR, thereby protecting the HuR protein from polyubiquitination- and proteasome-dependent degradation. HuR stabilized *STAT3* mRNA and upregulated the levels of basal and phosphorylated STAT3 protein, persistently activating STAT3 signaling. Our research revealed that the lncRNA GMDS-AS1 and its direct target HuR constitutively activate STAT3/Wnt signaling and promote CRC tumorigenesis, the GMDS-AS1-HuR-STAT3/Wnt axis is a therapeutic, diagnostic and prognostic target in CRC.

## Introduction

Colorectal cancer is one of the most common cancers and is the third leading cause of morbidity globally; nearly 10% of malignant cancer cases and deaths are categorized as a CRC [1]. CRC incidence remains high in transitioned countries; moreover, it has increased substantially in developing countries due to dietary changes and lifestyle modification while limited medical service results in a higher mortality in transitioning countries. The underlying causes of this high disease burden need to be further analyzed [1]. The many CRC patients and limitations of current screening methods and therapeutics make research into new biomarkers and drug targets an urgent task.

Inflammatory bowel disease can increase the occurrence of adenoma and promote higher-grade carcinoma formation, which leads to the initiation of CRC [2]. Aberrantly activated inflammatory signaling, such as that in the NF-κB, IL-6/STAT3 and Cox-2 pathways, caused a higher DNA mutation burden and enhanced oncogene transcription. These pathways promote the survival, proliferation and chemoresistance of CRC cells by evading apoptosis, effectively enhancing CRC cell invasion, angiogenesis and metastasis [3].

Long noncoding RNAs (lncRNAs) are defined as RNAs with a length >200 bp lacking protein-coding potential. Deep sequencing of the tumor transcriptome has revealed numerous lncRNAs that are abnormally expressed in a variety of malignant tumors, including CRC [4], and multiple biological functions of lncRNAs in CRC progression have been identified [5, 6]. Many studies have elucidated the regulatory function of CRC-related lncRNAs at the transcriptional [7], posttranscriptional [8], translational [9] and posttranslational levels [10] through interactions with nuclear acids and proteins [11]. Several CRC-associated lncRNAs are reported to be closely related to inflammatory signaling pathways, especially the IL-6/STAT3 pathway. The lncRNA ITIH4-AS1 recruits the FUS protein and facilitates the nuclear entry of phosphorylated-stabilized signal transducer and activator of transcription 3 (p-STAT3) dimers [12]. The classical anti-inflammatory drug aspirin upregulates the transcription of the lncRNA OLA1P2, which blocks p-STAT3 homodimer formation [13].

Although the relationship between ncRNAs and IBD is gradually becoming clear [14], most of the research into this relationship remains focused on changes to the inflammatory tumor microenvironment induced by microRNAs. The lncRNA GMDS-AS1 was first described as a potential biomarker for assessing the prognosis of hepatocellular carcinoma [15]. However, the critical role played by GMDS-AS1 in CRC remains unknown and warrants further exploration.

Persistent activation of NF-κB signaling and STAT3 signaling links inflammation and the development and progression of CRC [16, 17]. We have shown that the miR221/222-mediated positive feedback loop maintains the persistent activation of NF-κB and STAT3 by binding to PDLIM2 in CRCs [18]. The links bridging the persistent activation of inflammatory signals and other CRC-promoting signals, such as Wnt signaling, need to be elucidated.

In this study, we identified a novel CRC-promoting lncRNA GMDS-AS1 induced by IL-6 and Wnt3a. GMDS-AS1 directly interacts with human antigen R (HuR) and blocks its ubiquitination/proteasome-dependent degradation. The IL-6/Wnt3a-GMDS-AS1-HuR complex consequently increases total and p-STAT3 through HuR-mediated STAT3 mRNA stabilization and activates several important signaling pathways that promote CRC, including Wnt signaling. IL-6/Wnt3a-GMDS-AS1-HuR-STAT3/Wnt triggers a series of oncogenic biological functions, including a decreased apoptosis rate, an accelerated proliferation rate and stem cell-like phenotype acquisition by CRC cells.

## Results

### IL-6 induced high GMDS-AS1 expression in CRC, which indicated a poor prognosis

IL-6 is a well-characterized proinflammatory ligand that can initiate Jak-STAT signaling to promote tumor progression [19]. To identify inflammation-related lncRNAs in CRC, we used an lncRNA chip with IL-6 treated HCT116 cells, and the lncRNA expression profiles were compared. The data showed that the expression of 117 lncRNAs was significantly upregulated, and 7 of these lncRNAs were selected for functional screening (Fig. S1A and Supplementary Table 1). We designed two short hairpin RNAs (shRNAs) to target each candidate lncRNA and assessed the effect of knocking down candidate lncRNA expression on the proliferation of CRC cells (data not shown). Among the seven candidate lncRNAs, we found that lncRNA GDP-mannose-4,6-dehydratase-antisense 1 (GMDS-AS1) exerted the greatest inhibitory effect. GMDS-AS1 is located on chromosome 6p25.3-p25.2 and is head-to-head transcribed in the direction opposite that of the GMDS gene (Fig. S1B). Full-length GMDS-AS1, consisting of 1472 bp, was determined by 5’ and 3’ rapid amplification of cDNA ends (RACE) (Fig. S1C, D). The open reading frame finder, CPC2/CPAT [20, 21] and PhyloCSF score [22] indicated that GMDS-AS1 has a lncRNA-like sequence with little protein-coding potential (Fig. S2A–C). Additionally, GMDS-AS1 was mainly (~88%) expressed in the nucleus of CRC cells, while some (~12%) GMDS-AS1 remained in the cytoplasm (Fig. S2D). The LncATLAS database [23] indicated a similar cytoplasmic-nuclear localization pattern for GMDS-AS1 in multiple human cancer/stem cell lines (Fig. S2E).

**Fig. 1:**
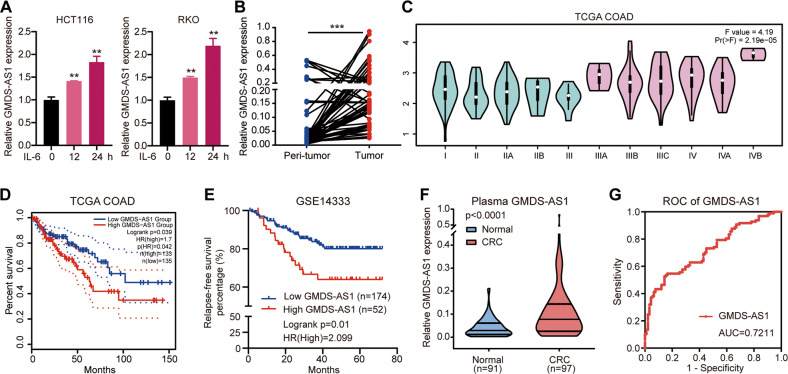
GMDS-AS1 indicates poor clinical outcomes in CRC. **A** Validation of GMDS-AS1 expression induced by IL-6 (10 ng/ml) through quantitative real-time PCR. **B** GMDS-AS1 RNA levels in 50 pairs of CRC tissues and adjacent normal tissues were detected by qPCR. ***p < 0.001. **C** Relative GMDS-AS1 RNA expression in 478 CRC patients in the TCGA COAD dataset cohort. High GMDS-AS1 positively correlated with later TNM stages. **D** Kaplan–Meier analyses showing the correlation between GMDS-AS1 RNA levels and overall survival in 268 TCGA COAD patients. The CRC patients were stratified on the basis of the median GMDS-AS1 RNA level (log-rank test). **C**, **D** The figure was generated with GEPIA2. **E** Kaplan–Meier analyses of the correlation between GMDS-AS1 RNA levels and relapse-free survival in 226 CRC patients in the Tumor Colon-Sieber-290-MAS5.0-u133p2 cohort (GSE14333). Patients were stratified on the basis of the upper quartile GMDS-AS1 value. **F** The expression levels of GMDS-AS1 were measured in CRC plasma samples from 97 CRC patients and 91 patients with gastrointestinal inflammation serving as control individuals by qPCR. The Mann–Whitney *U* test was performed to determine statistical significance. **G** Receiver-operating characteristic (ROC) curves and the corresponding area under the curve (AUC) values for the plasma GMDS-AS1 data presented in **F**.

Subsequently, we validated the significant GMDS-AS1 expression induced by IL-6 stimulation in HCT116 and RKO cells (Fig. 1A). GMDS-AS1 expression levels were markedly increased in the 50 CRC tissues compared with those in paired peri-tumor specimens (Fig. 1B). By analyzing GMDS-AS1 expression levels and clinicopathological feature data in the 50 CRC tissues and in The Cancer Genome Atlas (TCGA) colon adenocarcinoma (COAD) dataset [24], we found that late-TNM-stage CRC patients showed notably higher GMDS-AS1 expression (Fig. 1C and Table S6). The TCGA dataset also suggested that the patients in the high GMDS-AS1 expression group experienced a shorter overall survival time (Fig. 1D) and disease-free survival time (Fig. S1E) than those in the low GMDS-AS1 expression group. A similar relationship between high GMDS-AS1 expression and short relapse-free survival was identified in another cohort (Tumor Colon-Sieber-290, GSE14333) (Fig. 1E). These data indicated that high GMDS-AS1 expression was substantially and positively correlated with CRC progression and poor prognoses. LncRNAs are reported to be potential diagnostic and predictive CRC biomarkers with acceptable sensitivity and specificity [25, 26]. Furthermore, GMDS-AS1 may serve as a potential CRC diagnostic biomarker. Plasma GMDS-AS1 expression levels in 97 CRC patients and 91 patients with gastrointestinal inflammation were measured. The results revealed that GMDS-AS1 in the CRC plasma samples was substantially higher than that in the control samples (Fig. 1F), and the area under the curve demonstrated fair specificity and sensitivity (Fig. 1G). In conclusion, these results revealed that GMDS-AS1 is an inflammation-related lncRNA and might play a key role in CRC carcinogenesis.

### GMDS-AS1 contributes to proliferation and inhibits apoptosis in CRC cells in vitro and in vivo

**Fig. 2:**
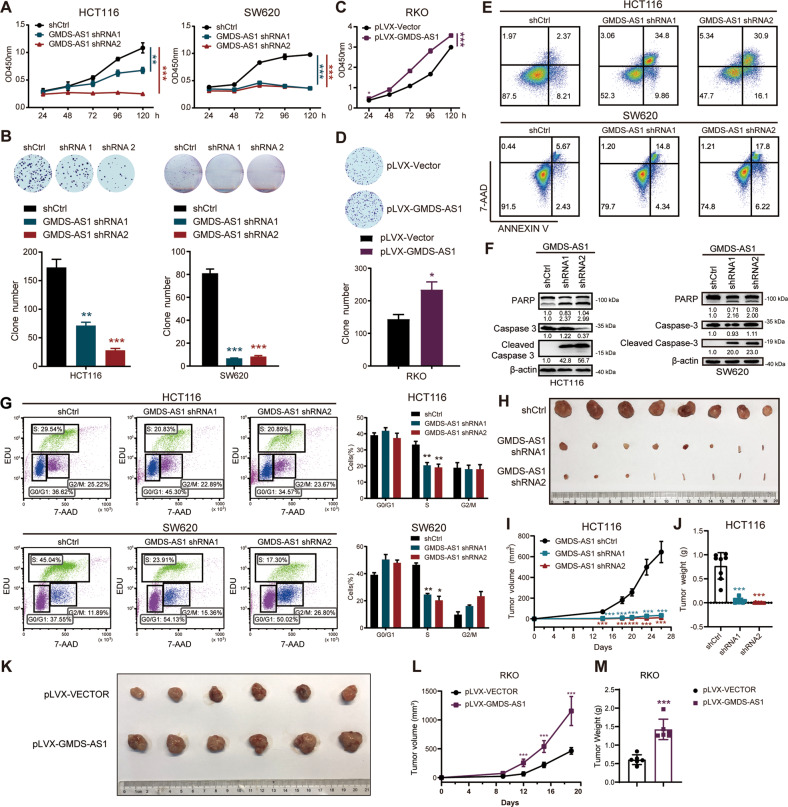
GMDS-AS1 promotes CRC cell proliferation and inhibits CRC cell apoptosis in vitro and in vivo. **A** Cell viability was determined by CCK-8 assay with HCT116 and SW620 cells stably transduced with control shRNA (shCtrl) or GMDS-AS1 shRNAs (shRNA1 or shRNA2). **B** Colony formation assays with HCT116 and SW620 cells stably transduced with control shRNA or GMDS-AS1 shRNAs. Above, representative images; below, quantification. **C** CCK-8 assays of control and stable GMDS-AS1-expressing RKO cells. **D** Colony formation assays with control and stable GMDS-AS1-expressing RKO cells. Above, representative images; below, quantification. **E** The percentage of apoptotic HCT116 and SW620 cells was estimated by flow cytometry using APC-labeled Annexin V and 7-AAD staining. Representative result based on three independent experiments. **F** Immunoblot analysis of the apoptosis hallmarks cleaved PARP and Caspase-3 in HCT116 and SW620 cells stably transduced with a control shRNA or GMDS-AS1 shRNA. **G** Cell cycle evaluation of HCT116 and SW620 cells stably transduced with control shRNA or GMDS-AS1 shRNA by EdU/7-AAD double-staining with flow cytometry. Left: flow plots. Right: quantification. **H** Representative image of xenograft tumors excised from nude mice subcutaneously injected with control or GMDS-AS1 KD HCT116 cells (*n* = 8). The small pieces of mouse tails represent the mice that failed to form tumors. **I**, **J** Tumor volume and tumor weight of the mice shown in **H** were measured. **K** Representative image of xenograft tumors excised from nude mice subcutaneously injected with control or GMDS-AS1 OE RKO cells (*n* = 6). **L**, **M** Tumor volume and tumor weight of the mice shown in **K** were measured. **A**–**D** Values are expressed as the means ± SEM, *n* = 3. ***p < 0.001, **p < 0.01, *p < 0.05 by two-tailed Student’s *t* test.

The expression of GMDS-AS1 in four CRC cell lines was examined and the GMDS-AS1 abundance was SW620, HCT116, SW480 and RKO in sequence (Fig. S3A). We next constructed lentivirus-infected stable GMDS-AS1-knockdown (KD) HCT116, SW620 and RKO cell lines using two independent shRNAs (GMDS-AS1 shRNA1 and shRNA2) and a nontarget control shRNA (shCtrl). The GMDS-AS1 level in KD CRC cells decreased substantially (Fig. S3B). The silencing of GMDS-AS1 led to a dramatic decrease in CRC cell line growth rates, as measured by a cell counting kit-8 (CCK-8) assay (Figs. 2A and S3D). The colony formation ability was similarly suppressed in the GMDS-AS1-KD CRC cell lines (Figs. 2B and S3E). Consistent with the aforementioned results, stable GMDS-AS1-overexpressing (OE) RKO cells (Fig. S3F) showed an accelerated proliferation rate and formed denser colonies (Fig. 2C, D). Additionally, we performed fluorescence-activated cell sorting (FACS) with Annexin V/7-amino-actinomycin D (7-AAD) double staining and found that the GMDS-AS1-KD CRC cell apoptosis rate was increased (Fig. 2E). To verify this effect, immunoblot analysis confirmed that the cleaved caspase-3 and cleaved PARP expression levels were upregulated in the GMDS-AS1-KD HCT116 and SW620 cells (Fig. 2F). EdU/7-AAD staining and FACS showed that silencing GMDS-AS1 reduced the percentage of CRC cells in the S phase of the cell cycle (Fig. 2G). To estimate the pro-CRC growth effect of GMDS-AS1 in vivo, we subcutaneously injected GMDS-AS1-KD HCT116 cells into BALB/c nude mice. Compared to the control group, the GMDS-AS1 KD group showed dramatic tumor shrinkage (Fig. 2H). The volume and weight of the GMDS-AS1-KD xenograft tumors were markedly reduced (Fig. 2I, J). Similarly, GMDS-AS1 OE RKO cells exhibited a higher proliferative ability than control cells in vivo (Fig. 2K–M). Notably, these experiments suggest that GMDS-AS1 facilitates CRC cell avoidance of apoptosis in vitro and promotes CRC cell proliferation in vitro and in vivo.

### GMDS-AS1 promotes stem cell-like properties in CRC cells

We next evaluated whether GMDS-AS1 modulates CRC cell stemness. GMDS-AS1 depletion markedly decreased HCT116 and RKO cell tumorsphere formation capacity (Fig. S4A). GMDS-AS1 KD also impaired the primary and secondary sphere formation of these CRC cells (Fig. S4B). Extreme limiting dilution analysis (ELDA) revealed that GMDS-AS1 KD HCT116 cells showed reduced sphere-initiating cell frequency in vitro (Fig. S4F) and tumor initiating frequency in vivo (Fig. S4G–I). With CSC markers staining and FACS, the results showed that silencing GMDS-AS1 reduced the generation of CD44^+^CD133^+^ and CD166^+^ cells in CRC cells. Additionally, we found that a series of CSC and EMT-related genes were expressed at significantly lower levels in GMDS-AS1-KD CRC cells in vitro (Fig. S4C) and in GMDS-AS1-KD HCT116 cells in vivo (Fig. S5L). Consistently, GMDS-AS1 overexpression visibly enhanced the sphere number and average diameter of HCT116 and RKO cells (Fig. S4D). Similarly, CSC and EMT-related gene mRNA expression levels were noticeably higher in GMDS-AS1-OE RKO cells in vitro and in vivo (Figs. S4E and S5M).

### GMDS-AS1 activates the Jak-STAT3 and Wnt signaling pathways in CRC cells

**Fig. 3:**
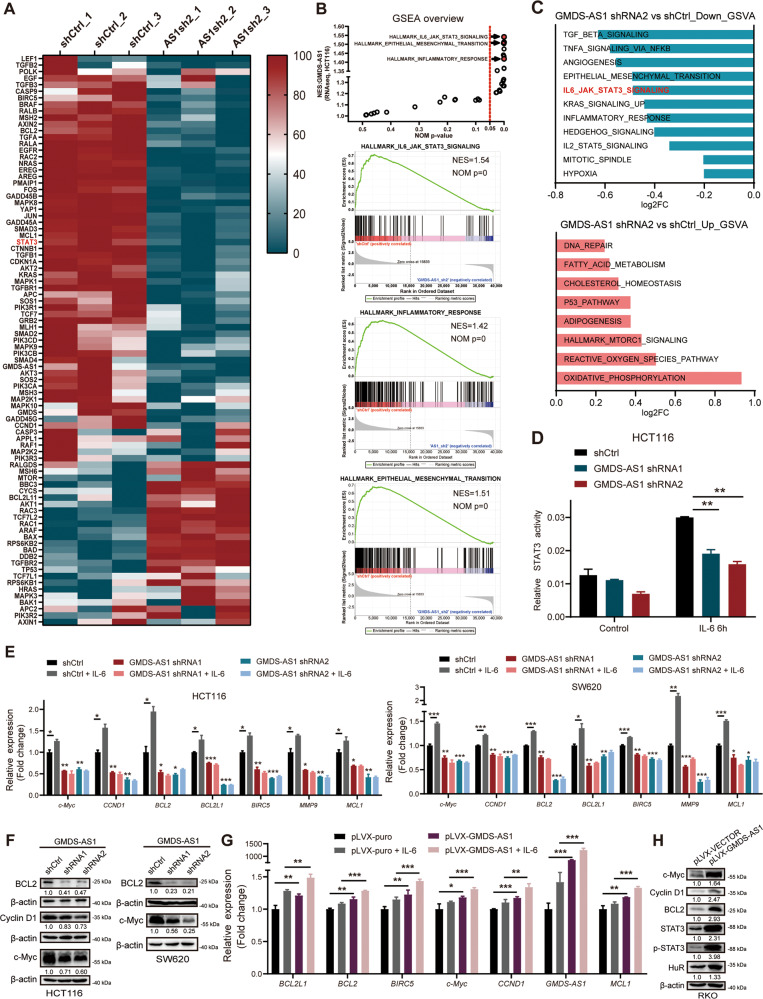
GMDS-AS1 activates the Jak-STAT3 signaling pathway in CRC cells. **A** CRC-related gene expression profiles of HCT116 cells stably transduced with the control shRNA (shCtrl) or GMDS-AS1 shRNA 2 plasmid. In the heatmap, the genes are shaded blue, white, or red to indicate low, intermediate, or high expression, respectively. **B** A GSEA of hallmark gene sets in the mRNA expression profile of HCT116 cells stably transduced with the shCtrl or GMDS-AS1 shRNA 2 plasmid. Three representative GSEA enrichment plots are shown. **C** A GSVA showing gene sets that were significantly enriched (*p* < 0.05), including the STAT3 signaling pathway. **D** Luciferase activity assays of the STAT3 signaling pathway were performed in HCT116 cells stably transduced with control or GMDS-AS1 shRNAs. **E** Relative mRNA expression levels of STAT3 target genes in IL-6 untreated/treated GMDS-AS1 control/KD HCT116 (above) and SW620 cells (below) were measured by qPCR. The cells were harvested after 24 h of vehicle or 10 ng/ml IL-6 treatment. **F** The expression levels of certain STAT3 downstream effector proteins in GMDS-AS1 control/KD HCT116 and SW620 cells were determined by western blotting. **G** Relative mRNA expression levels of STAT3 target genes in IL-6 untreated/treated RKO cells stably expressing a control or GMDS-AS1 plasmid were measured by qPCR. The cells were harvested after 24 h of vehicle or 10 ng/ml IL-6 treatment. **H** The expression levels of certain STAT3 downstream effector proteins in control and stable GMDS-AS1-expressing RKO cells were determined by western blotting. **D**, **E**, **G** Values are expressed as the means ± SEM, *n* = 3. ***p < 0.001, **p < 0.01, and *p < 0.05 by two-tailed Student’s *t* test. **E**, **G** 18S was the endogenous control. **F**, **H** β-actin was the endogenous control.

To elucidate the specific mechanism by which GMDS-AS1 promotes CRC initiation, we performed RNA-seq, and the transcriptome data were analyzed (GSE 205630). The RNA-seq results showed that GMDS-AS1 KD changed the expression levels of 2885 genes, and 865 genes were upregulated, while 2020 genes were downregulated (fold change ≥2, Supplementary Table 2). We identified a series of CRC-related oncogenes and tumor suppressor genes with observable expression changes; they included YAP1, CTNNB1, BAD, BAX, BCL2, IL6ST, MCL1, and particularly, STAT3 (Supplementary Table 3 and Fig. 3A). A gene set enrichment analysis (GSEA) (Fig. 3B) with 50 hallmark gene sets indicated that GMDS-AS1 KD exerted a strong influence on the IL-6-JAK-STAT3 signaling pathway, inflammatory response, epithelial-mesenchymal transition and other cellular functions. In addition, a gene set variation analysis (GSVA) revealed a variety of noteworthy GMDS-AS1-affected signaling pathways, including the Jak-STAT signaling pathway (Fig. 3C). Since GMDS-AS1 was induced by IL-6, we hypothesized that the Jak-STAT3 signaling pathway is activated by a feed forward mechanism initiated by GMDS-AS1 expression. Through STAT3 and TOP/FOP luciferase reporter assays, we confirmed that GMDS-AS1 inhibition (Fig. S3B, C) markedly suppressed IL-6–induced STAT3 activity and Wnt3a-induced β-catenin activity (Figs. 3D and S5D–G). After IL-6/Wnt3a stimulation, GMDS-AS1 was induced while GMDS-AS1-KD CRC cells showed a limited increase in STAT3/β-catenin target gene mRNA expression (Figs. 3E and S5H, I). A panel of STAT3 target genes expressed lower levels of mRNA (Figs. S3G and S5A, B, J) and protein (Fig. 3F) in response to GMDS-AS1 depletion in vitro and in vivo. Moreover, basal and cytokine-induced STAT3 target gene mRNA expression (Figs. 3G and S3H and S5C, K) and protein expression (Fig. 3H) were further elevated when GMDS-AS1 was overexpressed in vitro and in vivo.

### GMDS-AS1 increases STAT3 protein expression by stabilizing *STAT3* mRNA

**Fig. 4:**
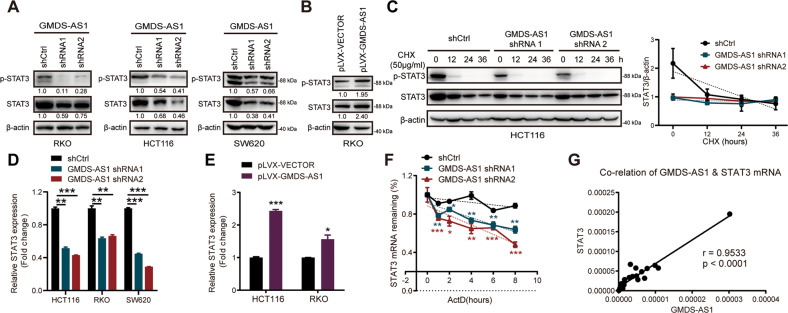
GMDS-AS1 increases STAT3 protein expression by stabilizing STAT3 mRNA. **A**, **B** The protein expression levels of p-STAT3 (Y705) and total STAT3 in RKO cells (**A**, left), HCT116 cells (**A**, middle) and SW620 cells (**A**, right) stably transduced with control or GMDS-AS1 shRNA were detected by immunoblotting. The same approach was used to measure p-STAT3 (Y705) and total STAT3 protein levels in RKO cells (**B**) stably expressing the control or GMDS-AS1 plasmid. **C** HCT116 cells stably transduced with control or GMDS-AS1 shRNA were treated with CHX (50 μg/ml) for the indicated times, and the protein expression levels of p-STAT3 (Y705) and total STAT3 were detected by immunoblotting (left). Densitometric analysis curve of total STAT3 protein levels (right). **A**–**C** β-actin was the endogenous control. **D**, **E** Relative mRNA expression levels of *STAT3* in GMDS-AS1 control/KD HCT116, SW620 and RKO cells were measured by qPCR (**D**). *STAT3* mRNA expression levels were detected in control and stable GMDS-AS1-expressing HCT116 and RKO cells (**E**). 18S was the endogenous control. **F** Relative mRNA expression levels of *STAT3* in HCT116 cells stably transduced with control or GMDS-AS1 shRNA after actinomycin D (ActD) (10 μM) treatment for the indicated times were measured by qPCR. **G** Correlation of GMDS-AS1 and *STAT3* mRNA expression levels in 23 CRC tissues as detected by qPCR. Pearson’s correlation analysis was performed to calculate the *R* and *p* values. **D**–**F** Values are expressed as the means ± SEM, *n* = 3. ***p < 0.001, **p < 0.01, and *p < 0.05 by two-tailed Student’s *t* test.

STAT3 protein plays a crucial role in the Jak-STAT3 signaling pathway. Once phosphorylated, the STAT3 dimer is translocated to the nucleus where it initiates the transcription of downstream oncogenes [19]. Overexpression and continuous activation of STAT3 have been observed in many types of chronic inflammation-related cancers, including CRC [27]. Knocking down GMDS-AS1 expression inhibited STAT3 phosphorylation and decreased basal STAT3 expression in HCT116, SW620 and RKO CRC cells (Fig. 4A). In contrast, GMDS-AS1-OE RKO cells exhibited higher p-STAT3 and STAT3 protein levels (Fig. 4B). We used cycloheximide (CHX) to block protein synthesis and observed that the half-life of the STAT3 protein was subsequently unaffected by GMDS-AS1 KD (Fig. 4C). A RIP assay suggested that the GMDS-AS1 and STAT3 proteins do not directly interact (Fig. S6A). Considering that GMDS-AS1 expression increased STAT3 protein expression but not by altering its degradation rate, we turned our attention to the *STAT3* mRNA expression level. Knocking down GMDS-AS1 decreased *STAT3* mRNA expression in three CRC cell lines (Fig. 4D). Consistent with these results, the *STAT3* mRNA expression level was enhanced in both GMDS-AS1-OE CRC cells (Fig. 4E) and GMDS-AS1-OE RKO cell spheres (Fig. S6B). We confirmed that GMDS-AS1 exerted no significant effect on the transcriptional level of STAT3, as the *STAT3* pre-mRNA expression levels in control and GMDS-AS1-KD CRC cells were similar (Fig. S6C). The RNA polymerase II inhibitor actinomycin D (ActD) was added to explore the key step leading to the elimination of *STAT3* mRNA expression. In HCT116 cells, repression of GMDS-AS1 resulted in faster *STAT3* mRNA degradation than that in the negative control (Fig. 4F). The data showed that GMDS-AS1 and *STAT3* mRNA expression was upregulated in CRC tissues compared to adjacent tissues (Fig. S6D) and that the *STAT3* mRNA expression levels were highly correlated with the lncRNA GMDS-AS1 expression levels (Figs. 4G and S6E). In summary, we found that GMDS-AS1 activated the Jak-STAT3 signaling pathway by stabilizing *STAT3* mRNA and then promoting STAT3 protein expression.

### GMDS-AS1 directly binds to HuR protein

**Fig. 5:**
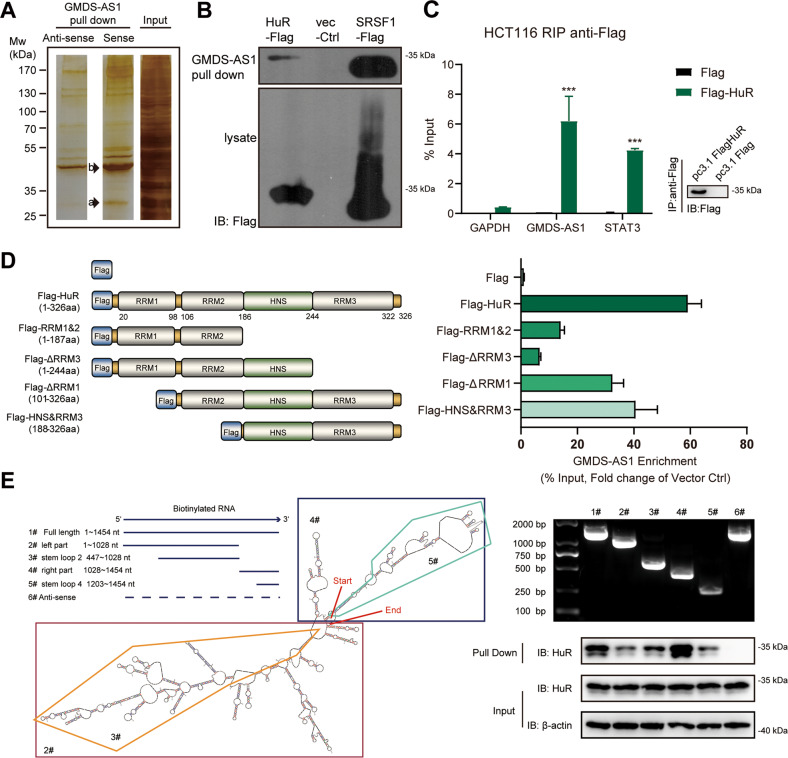
GMDS-AS1 binds to the HuR protein in CRC cells. **A** Biotinylated GMDS-AS1-sense and antisense probes were transcribed in vitro and incubated with HCT116 whole-cell lysates for use in RNA pull-down assays. After SDS–PAGE and silver staining, 28 kDa (arrow a) and 37 kDa (arrow b) GMDS-AS1-sense-specific bands, which repeatedly appeared in two independent assays, were excised and analyzed by mass spectrometry. **B** Streptavidin RNA pull-down assays and western blotting were performed, and the results validated the specific interactions of HuR or SRSF1 protein with GMDS-AS1. Representative results based on three independent experiments. **C** Plasmids expressing Flag-tag alone and Flag-HuR were transfected into HCT116 cells, and RIP experiments were performed using antibodies against Flag; qPCR was used to detect GMDS-AS1 and *STAT3* mRNA levels. *GAPDH* mRNA was used as the non-HuR target negative control. The values are expressed as the means ± SEM, *n* = 3. ***p < 0.001 by two-tailed Student’s *t* test. **D** Deletion mapping was performed to identify the GMDS-AS1 binding domain in HuR in HCT116 cells. HNS HuR nucleocytoplasmic shuttling sequence, RRM RNA recognition motif. Plasmids expressing a Flag-tag, Flag-tagged full-length HuR or Flag-tagged truncated HuR were transfected into HCT116 cells, and RIP experiments were performed using antibodies against Flag. qPCR was performed to detect the GMDS-AS1 enrichment levels. **E** Western blotting of HuR in streptavidin RNA pull-down samples by full-length biotinylated-GMDS-AS1 (1#), truncated biotinylated-GMDS-AS1 RNA motifs (2–5#) or biotinylated GMDS-AS1 antisense. The secondary structure of GMDS-AS1 was analyzed by Sfold (https://sfold.wadsworth.org/cgi-bin/srna.pl).

**Fig. 6:**
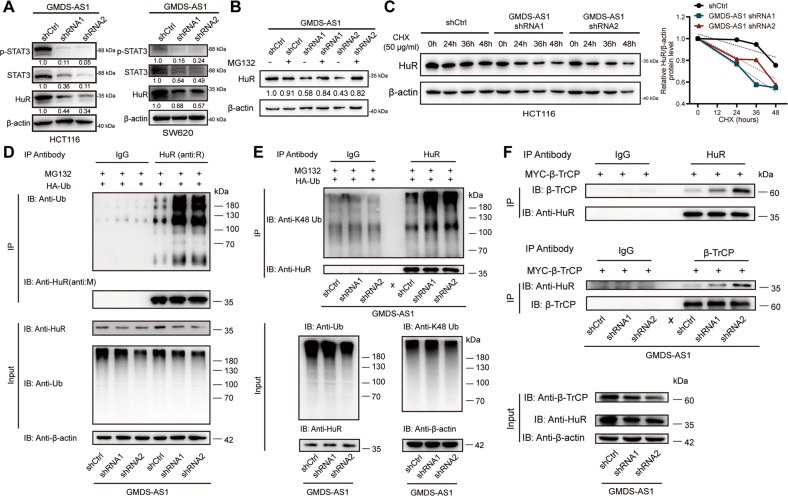
GMDS-AS1 blocks ubiquitination-dependent proteasomal degradation of HuR. **A** The protein levels of HuR, p-STAT3 (Y705) and total STAT3 in HCT116 cells (left) and SW620 cells (right) stably transduced with control or GMDS-AS1 shRNA were detected by immunoblotting. β-actin was the endogenous control. **B** GMDS-AS1 control/KD HCT116 cells were treated with MG132 (50 μg/ml) or dimethyl sulfoxide (DMSO) for 8 h, and the protein level of HuR was detected by immunoblotting. Densitometric analyses were performed with ImageJ software. The relative level of HuR protein expression was calculated and is labeled below the charts (normalization on the basis of the β-actin expression level). **C** GMDS-AS1 control/KD HCT116 cells were treated with CHX (50 μg/ml) for the indicated times, and the protein expression level of HuR was detected by immunoblotting (left). Densitometric analysis curve of the HuR protein expression levels (right). β-actin was the endogenous control. **D**, **E** Immunoprecipitation (IP) experiments were performed to detect the polyubiquitination and K-48 polyubiquitination levels of HuR after GMDS-AS1 expression was knocked down in HCT116 cells. HCT116 cells stably transduced with control or GMDS-AS1 shRNA were transfected with hemagglutinin (HA)-ubiquitin (Ub)-expressing plasmids for 48 h. After treatment with 20 μM MG132 for 8 h, cell lysates were immunoprecipitated with either control IgG or an anti-HuR antibody and immunoblotted with an anti-ubiquitin antibody (**D**) and an anti-K48-Ub (**E**) antibody. HuR, Ub, K48-Ub and β-actin were the loading controls. **F** IP experiments were performed to detect an interaction between HuR and β-TrCP1 after GMDS-AS1 expression was knocked down in HCT116 cells. HCT116 cells stably transduced with control or GMDS-AS1 shRNA were transfected with MYC-β-TrCP-expressing plasmids for 48 h. The cell lysates were immunoprecipitated with control IgG and anti-HuR and anti-β-TrCP antibodies and immunoblotted with anti-HuR and anti-β-TrCP antibodies. HuR, β-TrCP and β-actin were the loading controls.

Normally, RNA–RNA interactions and subsequent RNA lifetime regulation are firmly orchestrated by multiple RNA-binding proteins (RBPs) [28]. We performed a biotinylated RNA pull-down assay followed by MS to identify GMDS-AS1-associated RBPs (Supplementary Table 4). Specific bands in the silver-stained SDS–PAGE gel representing ~37 and ~28 kDa proteins were more apparent in the GMDS-AS1 sense RNA pull-down sample, and through MS, the RBPs HuR and SRSF1 were identified, respectively (Fig. 5A). The HuR and SRSF1 proteins have been reported to exhibit the ability to mediate RNA stability [29, 30]. This combination of RBPs was validated by independent RNA pull-down assays (Fig. 5B). As SRSF1 depletion did not affect STAT3 expression (data not shown), we focused on the function of HuR. RIP assay confirmed that HuR engaged in a specific association with not only GMDS-AS1 but also *STAT3* mRNA (Fig. 5C). HuR contains three RNA recognition motifs (RRMs) that interact with the 3’ or 5’ untranslated region (UTR) of target RNA [31]. In RIP assays using full-length or truncated HuR proteins, RRM3 excision significantly repressed the GMDS-AS1 interaction with HuR (Fig. 5D), indicating that GMDS-AS1 was associated with the RRM3 region of the HuR protein. Based on the GMDS-AS1 structure predicted by Sfold [32], GMDS-AS1 truncated motifs were constructed, and a biotinylated RNA pull-down assay was performed. HuR interacts with the majority of GMDS-AS1 especially the 4# fragment (1028–1454 nt). Compared with the 4# fragment, the deletion of 1028–1203 nt (5# fragment, 1203–1454 nt) significantly reduced GMDS-AS1-HuR binding (Fig. 5E). Consistent with the binding site predicted by RBPmap [33] (Fig. 6S), the results demonstrated that HuR mainly interacted with the 1028–1203 nt fragment of GMDS-AS1.

### GMDS-AS1 blocks ubiquitination-dependent proteasomal degradation of HuR

RT–qPCR and immunoblot assays indicated that silencing GMDS-AS1 expression in CRC cells exerted no meaningful influence on HuR mRNA expression (Fig. S7A), but it profoundly suppressed HuR protein expression (Figs. 6A and S6I, J). GMDS-AS1-OE RKO cells showed unchanged HuR mRNA expression levels (Fig. S7B) and increased HuR protein expression levels (Figs. 3H and S6H). Notably, this change in GMDS-AS1 mRNA expression induced up- or downregulated HuR protein expression, usually accompanied by corresponding basal and p-STAT3 protein level changes (Figs. 3H and 6A). From this, we hypothesized that this effect was based on a posttranslational modification. By MG132 treatment, we found that the suppression of endogenous HuR accumulation caused by GMDS-AS1 KD was relieved by blocking the proteasomal degradation pathway (Fig. 6B). Treatment with CHX led to a reduction in the lifespan of the HuR protein in GMDS-AS1-KD CRC cells (Fig. 6C). HuR can be ubiquitinated and eliminated via the proteasome, and beta-transducin repeat-containing protein (β-TrCP) has been identified as the major E3 ubiquitin ligase [34–36]. The GMDS-AS1-KD cells exhibited noticeably more abundant total and K48 endogenous HuR ubiquitination in IP experiments (Fig. 6D, E). An IP assay showed that knocking down GMDS-AS1 expression clearly promoted the HuR and β-TrCP interaction (Fig. 6F). These experimental findings indicated that GMDS-AS1 protected the HuR protein from E3 ligase β-TrCP-assisted ubiquitination and thus prevented HuR proteasome-mediated degradation.

### HuR functionally mediates GMDS-AS1 maintenance of stable *STAT3* mRNA expression

**Fig. 7:**
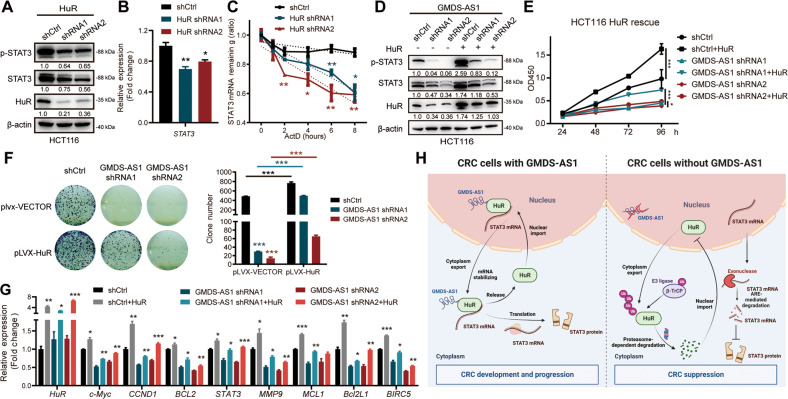
HuR functionally mediates GMDS-AS1 to maintain stable STAT3 mRNA expression. **A** The protein levels of HuR, p-STAT3 (Y705) and total STAT3 in HCT116 cells stably transduced with control or HuR shRNA were detected by immunoblotting. β-actin was the endogenous control. **B** Relative mRNA expression levels of *STAT3* in HuR control/KD HCT116 cells were measured by qPCR. **C** Relative mRNA expression levels of *STAT3* in HuR control/KD HCT116 cells after ActD (10 μM) treatment for the indicated times were measured by qPCR. **D** The protein expression levels of HuR, p-STAT3 (Y705) and total STAT3 in GMDS-AS1 control/KD HCT116 cells with or without HuR overexpression were detected by immunoblotting. **E** CCK-8 assays of control and GMDS-AS1-KD HCT116 cells with or without HuR overexpression. **F** Colony formation assays of control and GMDS-AS1-KD HCT116 cells with or without HuR overexpression. Left, representative images; right, colony number quantification. **G** Relative mRNA expression levels of STAT3 target genes in GMDS-AS1 control/KD HCT116 cells with or without HuR overexpression were measured by qPCR. 18S was the endogenous control. **B**, **C**, **E**–**G** Values are expressed as the means ± SEM, *n* = 3. ***p < 0.001, **p < 0.01, and *p < 0.05 by two-tailed Student’s *t* test. **H** Schematic illustration showing the GMDS-AS1-HuR-STAT3 axis and its regular function in CRC oncogenesis. In the presence of GMDS-AS1, HuR binds to STAT3 mRNA in the nucleus, and this complex is then transported to the cytoplasm. HuR prevents STAT3 mRNA from rapid AU-rich element (ARE)-mediated degradation; this protective effect maintains high STAT3 protein expression levels and promotes CRC progression. When GMDS-AS1 is depleted, more HuR proteins are ubiquitinated by the E3 ligase β-TrCP and eliminated via the proteasome pathway. In this case, the STAT3 protein level is dramatically decreased, and CRC tumorigenesis is blocked (Created with BioRender.com).

Because GMDS-AS1 blocks HuR degradation and since HuR is a well-characterized RBP, we assumed that the ability of GMDS-AS1 to stabilize *STAT3* mRNA is mediated by HuR. We validated the HuR and *STAT3* mRNA association by performing RIP assays (Fig. 5C). Specific shRNAs were used to stably knockdown HuR expression in HCT116 cells (Fig. S7C), and markedly reduced p-STAT3 and STAT3 protein expression levels, as well as *STAT3* mRNA expression levels, were observed in HuR-KD cells (Fig. 7A, B). Notably, HuR KD repressed STAT3 target gene expression (Fig. S7D). HuR did not alter the STAT3 protein half-life but lowered *STAT3* mRNA stability, similar to GMDS-AS1 (Figs. S7E, F and 7C). Exogenous HuR introduction notably abolished the downregulation of p-STAT3 and STAT3 expression caused by GMDS-AS1 KD (Fig. 7D). Similarly, the inhibited STAT3 target gene mRNA expression induced by GMDS-AS1 KD was reversed after exogenous HuR was expressed (Fig. 7G). HuR overexpression also significantly rescued CRC cell proliferation and colony formation that had been suppressed by GMDS-AS1 KD (Fig. 7E, F). The TCGA COAD dataset revealed a strong correlation between HuR and *STAT3* mRNA expression levels (Fig. S7G), corroborating the importance of HuR in stabilizing *STAT3* mRNA expression. These results help to explain the critical role played by HuR in GMDS-AS1-induced STAT3 signaling pathway activation and the subsequent CRC oncogenic effects.

## Discussion

Our findings confirmed that GMDS-AS1 is a promising CRC diagnostic and prognostic biomarker and is directly involved in CRC pathogenesis. GMDS-AS1 expression was enhanced in CRC tissues and exhibited an upward trend with advancing clinical tumor stage. By analyzing two independent cohorts, we validated that high GMDS-AS1 expression indicated a poor prognosis in CRC. Moreover, higher plasma GMDS-AS1 levels distinguished these CRC patients in the early tumor stage from noncancer control patients. GMDS-AS1 shows high CRC tumorigenicity, as cell viability, cell proliferation and stem cell-like phenotype changes were observed in functional assays.

Interestingly, IL-6-induced GMDS-AS1 expression leads to a feedforward effect in the activated STAT3 signaling pathway. These data further highlight the inextricable link between GMDS-AS1 and the pro-CRC inflammatory STAT3 signaling pathway. STAT3 induces the expression of downstream oncogenes, which cause pro-oncogenic effects in many ways, including but not limited to antiapoptotic effects, proliferation enhancement, stem cell phenotype acquisition, EMT and angiogenesis [27, 37, 38]. When GMDS-AS1 was depleted, the phosphorylated and basal STAT3 protein levels were considerably decreased, and STAT3 transcriptional activity and STAT3 target gene expression were suppressed These findings were unexpected because GMDS-AS1 did not directly bind to STAT3 to regulate its protein level, while most STAT3 regulator lncRNAs change the phosphorylation status of STAT3, control its dimer formation or alter its cytoplasmic-nuclear translocation dynamics [12, 13, 39]. Thus, we presumed that GMDS-AS1 upregulated STAT3 in pretranslational steps and, indeed, GMDS-AS1 may have stabilized *STAT3* mRNA. The high positive correlation between GMDS-AS1 and *STAT3* mRNA expression in the clinical samples proved this point of view and the critical role GMDS-AS1 plays in CRC STAT3 signaling.

In previous studies, GMDS-AS1 was described as an RNA sponge [40]. Here, we proved that GMDS-AS1 in CRC maintains excessive *STAT3* mRNA levels by directly binding to and stabilizing the RBP HuR. Studies have revealed that HuR combines with 3’ UTR AU-rich elements in target mRNAs in the nucleus, and HuR-mRNA conjugates are cotransported to the cytoplasm, with HuR stabilizing target mRNAs in this process and then moving back to the nucleus [41–44]. HuR has been shown to be regulated by noncoding RNAs and to contribute to CRC tumorigenesis by stabilizing β-catenin mRNA [35, 45, 46]. Similar to GMDS-AS1, HuR is mainly distributed within cell nuclei, but a fraction of HuR localizes to the cytoplasm. Herein, we showed the GMDS-AS1-HuR association and found that E3 ligase β-TrCP-mediated HuR ubiquitination and degradation were blocked by GMDS-AS1. According to MS analysis, GMDS-AS1 did not bind to β-TrCP. However, previous research has suggested that the β-TrCP-binding site is located in HuR RRM3 [34]. Interestingly, GMDS-AS1 interacts with RRM3 in HuR. Therefore, we speculate that GMDS-AS1 may competitively inhibit the β-TrCP–HuR association, and the details of this mechanism remain to be intensively explored.

Although HuR has been reported to stabilize the mRNA of many genes including β-catenin, no research has described that HuR can stabilize *STAT3* mRNA. In a previous study, 4810 HuR-binding RNAs were identified, including *STAT3* mRNA [47]. Moreover, the association between *STAT3* mRNA and HuR was verified by another independent study [48]. We proved that HuR RRM3 is the *STAT3* mRNA binding region (Fig. S6F). Since RRM3 plays a central role in the RNA-stabilizing process [43, 49], it is reasonable to think that our results revealed HuR maintenance of *STAT3* mRNA stability. Rescue assays suggested that GMDS-AS1 stabilizes *STAT3* mRNA expression mediated by HuR. Furthermore, *STAT3* mRNA expression was highly correlated with HuR mRNA expression in the TCGA COAD cohort. Exogenous HuR introduction also clearly abolished the restriction of CRC cell viability and proliferation caused by GMDS-AS1 KD.

The RNA-seq data indicated that GMDS-AS1 activates not only STAT3 signaling but also other CRC-related signaling pathways, such as the Wnt (Fig. S5E–I) and Hippo pathways, making them potential GMDS-AS1 targets. Based on previous reports, we speculate that HuR could maintain β-catenin mRNA and thus mediate GMDS-AS1-induced Wnt pathway activation. The fact that GMDS-AS1 was induced by both IL-6 (Figs. 1A and 3G) and Wnt-3a (Fig. S5H, I) means that there is one GMDS-AS1/HuR-mediated positive feedback to maintain persistent activation of STAT3 and Wnt signaling, and the GMDS-AS1-HuR-STAT3/Wnt axis connects the inflammation signal to the development and progression of CRC. Notably, the reversal induced by exogenous HuR was partial, which suggested that other underlying mechanisms are involved in the CRC oncogenic effect resulting from GMDS-AS1, which is worth exploring in the future.

## Conclusions

Ultimately, for the first time, we described that the lncRNA GMDS-AS1 is overexpressed and exhibits a tumor-promoting function in CRC. GMDS-AS1 was induced by IL-6, which activated the STAT3 signaling pathway, thereby enhancing malignant phenotype acquisition by CRC cells in vitro and in vivo. GMDS-AS1 physically interacted with HuR protein, preventing the ubiquitination–proteasome degradation of HuR and stabilizing *STAT3* mRNA (Fig. 7H). The characterization of the IL-6/GMDS-AS1/HuR/STAT3 axis provides a new perspective for understanding the pathogenesis of colitis-associated CRC and a novel diagnostic and therapeutic target in CRC.

## Materials and methods

### In vivo tumor xenografts

Specific-pathogen-free (SPF) grade BALB/C nude mice (6-week-old males) were purchased from Shanghai SLAC Laboratory Animal Company and randomly allocated to groups. All procedures were performed with the signed ethics approval of the Institutional Animal Care and Use Committee of Shanghai Institute of Nutrition and Health, Chinese Academy of Sciences, IACUC issue No. SIBS-ZXR-2019-2. Nude mice were randomly grouped. A total of 1 × 10^6^ lentiviral-transduced HCT116 and 3 × 10^6^ of RKO CRC cells were resuspended in 100 μl of sterile PBS and injected subcutaneously into nude mice. The major tumor axis (length, *L*) and minor axis (width, *W*) were measured twice per week, and the tumor volume was calculated with the formula *V* = 0.5 × *L* × *W*^2^. All mice were sacrificed from 4 to 5 weeks after the injection, and the tumors were removed and weighed.

### Human CRC tissue

All frozen CRC and corresponding adjacent noncancerous colon tissues were obtained from the tissue bank of Shanghai Institute of Nutrition and Health, Chinese Academy of Sciences. The ethics committee of the Institute approved this study, and all operations were performed following standard operating procedures authorized by the committee. Written informed consent was obtained from all patients.

### RNA immunoprecipitation (RIP) assay

CRC cells were cultured and transfected with Flag-tagged target plasmids in 10-cm dishes. Forty-eight hours after transfection, ultraviolet (UV) radiation cross-linking (300 mJ/cm^2^ at a 245-nm wavelength) was performed on ice. In total, 2 × 10^7^ CRC cells were collected using a cell scraper and resuspended in RIP lysis buffer containing RNase and proteinase inhibitors. The cell lysates were sonicated three times (at 10% maximal energy for 5 s each time) on ice and then stored at −80 °C. On day 2, unfrozen cells were centrifuged for 10 min at 12,000 rpm and 4 °C; the supernatant was collected; and “input” samples were obtained. Five micrograms of anti-Flag antibody was added to Protein A/G magnetic beads (B23202, Bimake), and this mixture was rotated at 4 °C for 2 h. Then, 100 μl of cell supernatant was mixed with Flag-labeled magnetic beads and 900 μl of RIP buffer (with RNase and proteinase inhibitors added), and the tubes containing this mixture were rotated at 4 °C overnight. The beads were separated in a magnetic field, and the supernatant was discarded. The beads were washed with RIP wash buffer four times, and RNAiso Plus (9109, TAKARA) was added to the samples to extract target protein-linked RNAs.

### RNA pull-down assay

Template preparation, a T7-Flash biotin-RNA transcription reaction and biotin-RNA purification were performed using an AmpliScribe T7-Flash biotin-RNA transcription kit (ASB71110, Epicenter) following the manufacturer’s protocol. In total, 1 × 10^8^ CRC cells were collected using a cell scraper and resuspended in RIP lysis buffer 2, to which 1 mM RNase and 2 mM proteinase inhibitors were added. Biotin-RNA was mixed with sonicated cell lysates and rotated at room temperature for 1.5 h. Then, 40 μl of streptavidin Dynabeads (88816, Pierce) was added, and the mixture was rotated at room temperature for another 1.5 h. The beads were washed with RIP buffer 2 (with 0.5% sodium deoxycholate added), and 5× SDS–PAGE loading buffer was added. Regular SDS–PAGE sample preparation and western blot procedures were performed. For mass spectrometry analysis, SDS–PAGE gels were silver-stained with a fast silver stain kit (P0017S, Beyotime).

### Cell culture

HCT116, SW620, and RKO human colorectal cancer and HEK-293T human cells were purchased from American Type Culture Collection (ATCC). Mycoplasma contamination testing was performed and the cells were proved to be mycoplasma free. All the cell lines were cultured under the recommended culture conditions, and the recommended base medium (McCoy’s 5a Medium for the HCT116 cells, Leibovitz’s L-15 Medium for the SW620 cells and Dulbecco’s modified Eagle’s medium for the RKO and HEK-293T cells) with fetal bovine serum (FBS) was added to a final concentration of 10%. The following reagents were used: MG132 (S1748, Beyotime), cycloheximide (CHX; C7698, Sigma), human interleukin-6 (IL-6; 200-06-20, Peprotech), and actinomycin D (ActD; HY-17559, MedChemExpress).

### Lentivirus packaging and infection

pMD2.G/psPAX2 plasmids and ViaFect (E4981, Promega) transfection reagent were used in the lentivirus packaging system. HEK-293T cell supernatants containing the lentiviruses were harvested and filtered 48 h after transfection. CRC cells were infected by lentivirus particles with 10 μg/ml polybrene (40804ES76, Yeasen).

### RNA extraction and quantitative real-time PCR (RT–qPCR)

RNAiso Plus (9109, TaKaRa) was used to extract total RNA from CRC cells according to the manufacturer’s protocol. One microgram of total RNA was reverse transcribed into cDNA using a PrimeScript RT reagent kit with gDNA Eraser (RR047A, TaKaRa). Quantitative real-time PCR (qRT–PCR) was performed with a QuantStudio 7 Flex Real-Time PCR System (4485701, ABI) and TB Green Premix Ex Taq (Tli RNaseH Plus) (RR420A, TaKaRa). Samples were tested in triplicate. The equation RQ = 2^−ΔΔCt^ was used to calculate the gene expression, and the data were normalized to those of the internal reference gene ACTB. The qRT–PCR primer sequences are listed in Supplementary Table 5.

### Western blot analysis

In total, 5 × 10^6^ CRC cells were harvested with a cell scraper, and RIPA lysis buffer (P0013B, Beyotime) was used to extract total protein. The concentration of total protein was measured with a bicinchoninic (BCA) protein assay kit (PC0020, Solarbio). Samples were mixed with 5x loading buffer and then heated at 100 °C for 10 min. Then, 25 μg of total protein was loaded onto SDS–PAGE gel, and a standard western blot procedure was performed. The separated proteins were transferred to PVDF membranes (IPVH00010, Millipore) and blocked with 5% nonfat milk. The membrane was incubated with primary antibodies overnight at 4 °C and then incubated with horseradish peroxidase (HRP)-conjugated secondary antibodies for 2 h at room temperature. The HRP-labeled proteins were visualized with an ECL kit (WBKLS0010, Millipore) and an automatic chemiluminescence image analysis system (5200 Multi, Tanon). All full-length uncropped western blots are provided in the Supplementary Material. The following antibodies were used: an anti-p-STAT3 antibody (Y705) (Cy6566, Abways), an anti-STAT3 antibody (sc-482, Santa Cruz), an anti-HuR rabbit polyclonal antibody (11910-1-AP, Proteintech), an anti-HuR mouse monoclonal antibody (mAb; 66549-1-Ig, Proteintech), an anti-β-actin antibody (30101ES60, Yeasen Biotech Co.), an anti-BCL2 antibody (12789-1-AP, Proteintech), an anti-C-MYC antibody (10828-1-AP, Proteintech), an anti-cyclin D1 antibody (sc-753, Santa Cruz), an anti-PARP antibody (#9542, CST), an anti-cleaved caspase-3 antibody (#9661, CST), an anti-caspase-3 antibody (#9662, CST), an anti-Flag antibody (F1804, Sigma), an anti-Ub (P4D1) mouse mAb (#3936, CST), an anti-K48-linkage Specific Polyubiquitin (D9D5) Rabbit mAb (#8081, CST), an anti-β-TrCP antibody (sc-390629, Santa Cruz), anti-rabbit IgG (sc-2027, Santa Cruz), anti-mouse IgG (sc-2025, Santa Cruz), peroxidase-conjugated goat anti-rabbit IgG (33101ES60, Yeasen Biotech Co.), and peroxidase-conjugated goat anti-mouse IgG (33201ES60, Yeasen Biotech Co.).

### Cell growth curve and colony formation assay

In vitro cell proliferation was detected with a cell counting kit-8 (CCK-8) assay kit (CK04, Dojindo). One hundred microliters of lentivirus-transduced CRC cells were seeded into 96-well plates (1 × 10^3^ per well in 3-well replicates). Ten microliters of CCK-8 reagent was added to the plate every 24 h, and the optical density was read at 450 nm 2.5 h later. Cell growth curves were drawn.

A total of 1.5 × 10^3^ lentivirus-transduced CRC cells (3-well replicates) were seeded into one well of a 6-well plate, and 3 ml of complete culture medium was added. The cells were cultured for 10 days, and the colonies were fixed with 4% formaldehyde. Crystal violet staining solution (C0121, Beyotime) was used to stain the colonies, and the colonies were counted.

### Plasmid construction and reagents

To knockdown target gene expression, gene-specific short hairpin RNAs (shRNAs) were cloned into a pLKO.1 puro plasmid (10878, Addgene) following the manufacturer’s guidelines. The full-length human GMDS-AS1 sequence and human HuR coding sequence were cloned with PrimeSTAR Max DNA Polymerase (R045A, TaKaRa) and cloned into a pLVX-IRES-puro plasmid (632183, Clontech). Flag-tagged HuR was cloned into a pcDNA3.1 (+) plasmid. All the shRNA sequences and clone primers are listed in Supplementary Table 5. The following reagents were used: NEBuffer 2 (B7002S, New England Biolabs), Age I (R3552S, NEB), EcoR I (R3101S, NEB), BamH I (FD-0054, Thermo Fisher), DNA ligation kit reagents (6022, TaKaRa), and TransScript First-Strand cDNA Synthesis SuperMix (AT301, TransGen Biotech).

### Sphere formation assay

A total of 2 × 10^3^ lentivirus-transduced CRC cells were seeded into one well of an ultralow adherent 24-well plate. The spheres were cultured in DMEM/F12 (SH30023.01, HyClone)-based tumorsphere medium for 7 days, and images were captured. The following tumorsphere medium recipe was used: 1/50 B27 supplement (#12587010, Invitrogen), 20 ng/ml epidermal growth factor (315-09, PeproTech), 10 ng/ml basic fibroblast growth factor (345-FG-025, R&D), and 4 μg/ml insulin (I9278, Sigma).

### In vitro and in vivo extreme limiting dilution assay (ELDA)

In vitro and in vivo ELDA was performed essentially as described in Agro et al. [50]. For in vitro ELDA, four doses of lentivirus-transduced HCT116 cells (1000, 100, 10, 1) were seeded into 12, 24, 60 and 96 wells of U-bottom non-tissue culture 96-well plates, respectively. Sphere formation was observed by a microscope 2 weeks later. For in vivo ELDA, three doses of lentivirus-transduced HCT116 cells (5 × 10^5^, 5 × 10^4^, 5 × 10^3^) mixed with matrigel (354248, Corning) were injected subcutaneously into 6-week-old BALB/c nude mice (male, *n* = 5 per group). Tumor volume were measured twice every week. The mice were sacrificed at 3 weeks post injection, and the tumor formation number and tumor weight were evaluated. The analysis of sphere/tumor initiating cell frequency and significance was calculated by ELDA software [51] (https://bioinf.wehi.edu.au/software/elda/).

### Fluorescence-activated cells sorting (FACS) analysis

CRC cell cycle and apoptosis analyses were performed by FACS. To detect the cell cycle, 1 × 10^5^ lentivirus-transduced CRC cells were seeded into one well of a 6-well plate and cultured for 24 h. The cells were labeled with 10 μM EdU for 2 h, digested with trypsin, fixed and stained with an EdU staining kit (BeyoClick EdU cell proliferation kit) with Alexa Fluor 488 (C0071, Beyotime) and 7-amino-actinomycin (7-AAD; 559925, BD) following the manufacturers’ protocols. For apoptosis analysis, 1.5 × 10^6^ lentivirus-transduced CRC cells were digested with trypsin and stained with Annexin-V (88-8007-74, Invitrogen) and 7-AAD (559925, BD) for 30 min in the dark. For CSC markers detection, 1.5 × 10^6^ lentivirus-transduced CRC cells were digested with trypsin and stained with CD44 (BioLegend, 397505), CD133 (566593, BD) and CD166 (BioLegend, 343905) for 30 min in the dark. A MoFlo Astrios Flow Cytometer (Beckman) was used for flow cytometry analyses.

### Luciferase reporter assay

A total of 1.5 × 10^4^ lentivirus-transduced CRC cells were seeded in one well of a 24-well plate and transfected with 300 ng of STAT3 reporter plasmid and 50 ng of the control Renilla vector (pRL-TK; Promega, Madison, WI) using ViaFect. Forty-eight hours after transfection, the cells were treated with 10 ng/ml IL-6 for 6 h. A dual-luciferase reporter system (#07311, Promega) was used to measure the luciferase activity in the cell lysates following the manufacturer’s protocol.

### RNA sequencing (RNA-seq)

Total RNA was extracted from lentivirus-transduced HCT116 cells and used for RNA-seq. An RNA library was constructed with an Illumina TruSeqTM RNA Sample Prep Kit, and Illumina NovaSeq 6000 was used by Shanghai Majorbio Biopharm Technology Co., Ltd. for sequencing. RNA expression levels were evaluated by Cufflinks (Version 2.2.1) and RSEM (Version 1.3.1) software. DESeq2 (Version 1.24.0) was used to perform differential expression analyses. As a reference tool, the Kyoto Encyclopedia of Genes and Genomes (KEGG) database (Version 2017.08) was used to identify GMDS-AS1-related pathways. The data were analyzed with the free online Majorbio Cloud Platform (www.majorbio.com).

### Immunoprecipitation (IP)

Lentivirus-transduced HCT116 cells were cultured and transfected with target plasmids in 6-cm dishes. For ubiquitination detection, 48 h after seeding transfection, the cells were treated with 20 μM MG132 for 6 h. A total of 1 × 10^7^ cells were washed with cold PBS twice, harvested by scraping and then centrifuged. The cells were lysed with co-IP buffer (0.5% Triton X-100, 20 mM HEPES, 150 mM NaCl, 12.5 mM glycerophosphate, 1.5 mM MgCl_2_, 2 mM EGTA, 1 mM PMSF, and a protease inhibitor cocktail solution) for 30 min and centrifuged for 15 min at 13,200 rpm and 4 °C. The supernatant fraction of the cell lysates was isolated, and a 10% input sample was collected. Two micrograms of IP antibody or IgG control was added to the remaining supernatant, and the mixture was incubated on a rotary shaker overnight at 4 °C. Then, 25 µl protein A/G magnetic beads (B23202, Bimake) were added the next day and incubated for an additional 4 h. Then, the beads were captured with a magnetic rack and washed 3 times with co-IP buffer. The beads were gently resuspended in 35 μl of 2× loading buffer and boiled at 100 °C for 5 min. A regular SDS–PAGE procedure was then performed.

### Statistical analysis

All the animal and cell experiments were biologically repeated three times. Sample size was determined based on the results of preliminary experiments. Two-sided Student’s *t* test or analysis of variance (ANOVA) was used and stated in the figure legend. All statistical tests were performed and graphed while the mean and standard deviation were calculated. Data are presented as the mean ± SEM. *p* value was labeled.

## Supplementary information

Supplementary figures and legends - final version

Figure S1

Figure S2

Figure S3

Figure S4

Figure S5

Figure S6

Figure S7

Table S1

Table S2

Table S3

Table S4

Table S5

Table S6

GMDS-AS1-HuR-STAT3 axis

Original Western Blot Image

Reproducibility Checklist

## Data Availability

The raw RNA-seq data were submitted to the GEO database. The GEO accession link is https://www.ncbi.nlm.nih.gov/geo/query/acc.cgi?acc=GSE205630. All other data are available from the corresponding author upon reasonable request.
