# Supplementary material for: LncGMDS-AS1 promotes the tumorigenesis of colorectal cancer through HuR-STAT3/Wnt axis
Source: Cell Death Dis. 2023 Feb 27;14(2):165. doi: 10.1038/s41419-023-05700-8 (PMC9970971; doi:10.1038/s41419-023-05700-8)
Supplement: Supplementary file 1 — Supplementary figures and legends - final version [file 41419_2023_5700_MOESM1_ESM.docx]

**Supplementary Figures and Legends**

**Figure S1. Characteristics of GMDS-AS1 in CRC**

**A.** Noncoding RNA expression in HCT116 cells treated without or with IL-6 for 24 hours. An Agilent Human lncRNA chip was used for detection. The noncoding RNAs are shaded blue and red in the heatmap to indicate low and high expression, respectively (fold change ≥ 2). **B.** Schematic loci diagram showing GMDS-AS1 and the neighboring gene GMDS on human chromosome 6p25.3-p25.2. **C, D.** Full-length GMDS-AS1 identification in HCT116 cells. **C**: PCR product images and sequencing results of 5’ rapid amplification of cDNA ends (RACE) and 3’ RACE; **D**: full-length sequence of GMDS-AS1. **E.** Kaplan–Meier analyses showing the correlation between GMDS-AS1 RNA levels and disease-free survival in 268 patients in The Cancer Genome Atlas (TCGA) colon adenocarcinoma (COAD) dataset cohort. CRC patients were stratified by GMDS-AS1 RNA level (log-rank test) on the basis of the median value. The figure was generated with the Gene Expression Profiling Interactive Analysis 2 (GEPIA2) tool.

**Figure S2. Protein-coding potential and subcellular localization identification of GMDS-AS1 in CRC**

**A.** Prediction of the GMDS-AS1 protein coding potential using the open reading frame (ORF) Finder (<https://www.ncbi.nlm.nih.gov/orffinder/>) **B, C.** Coding Potential Assessment Tool (CPAT, **B up**) (<http://lilab.research.bcm.edu/index.php>), Coding Potential Calculator 2 (CPC2, **B** **down**) (<http://cpc2.gao-lab.org/>) and PhyloCSF (**C**) tools were used to evaluate the protein coding potential of GMDS-AS1. *XIST* and *ANRIL* were the long noncoding RNA (lncRNA) controls, and *ACTB* and *GAPDH* were coding RNA controls. **D.** The distribution of GMDS-AS1 RNA in HCT116 and RKO cells (cytoplasmic, light blue; nuclear, pink). *ACTB* and *XIST* were the cytoplasmic and nuclear endogenous controls, respectively. As detected by qPCR, the values are expressed as the means ± SEM, n=3. **E.** GMDS-AS1 distribution in multiple human cell lines. The figure was generated with the LncATLAS tool (<https://lncatlas.crg.eu/>), and H19 and NEAT1 were the cytoplasmic and nuclear endogenous controls, respectively.

**Figure S3. GMDS-AS1 expression and knockdown/overexpression efficiency in CRC cells**

**A.** GMDS-AS1 RNA levels in different CRC cell lines. *ACTB* was the endogenous control and was detected by qPCR. **B.** Relative GMDS-AS1 RNA levels in HCT116, SW620 and RKO cells stably transduced with control short hairpin RNA (shCtrl) or GMDS-AS1 shRNA (shRNA-1 or shRNA-2). **C.** Relative GMDS-AS1 RNA levels in RKO cells transfected with control and GMDS-AS1 antisense oligonucleotide (ASO). **D.** Cell counting kit-8 (CCK-8) assays of RKO cells stably transduced with shCtrl and GMDS-AS1 shRNA. **E.** Colony formation assays with control and stable GMDS-AS1-knockdown (KD) RKO cells. Above, representative images; below, quantification. **F.** Relative GMDS-AS1 RNA levels in control and stable GMDS-AS1-expressing (plvx-GMDS-AS1-expressing) HCT116 and RKO cells. **G.** Relative GMDS-AS1 RNA levels in xenografted tumors from control and GMDS-AS1 shRNA2 HCT116 cells **H.** Relative GMDS-AS1 RNA levels in xenografted tumors from control and GMDS-AS1 OE RKO cells. **A-C, E, F**. Values are expressed as the means ± SEM, n=3. ***P< 0.001, **P < 0.01, and *P < 0.05 by two-tailed Student’s t test. **D, G, H.** Values are expressed as the means ± SEM, n=3. ***P< 0.001, **P < 0.01 by one-way ANOVA.

**Figure S4 GMDS-AS1 promotes stem cell-like properties in CRC cells *in vitro***

**A.** Sphere-forming assay with HCT116 and SW620 cells stably transduced with control short hairpin RNA (shCtrl) or GMDS-AS1 shRNA (shRNA-1 or shRNA-2). Left, representative images; right, the number of spheres formed by the indicated cells. Scale bar, 200 μm. **B.** Sphere-formation ratios of serial spheres formed by control and GMDS-AS1-knockdown (KD) HCT116 and RKO cells. **C.** Relative expression levels of CSC-related genes (CD133, SOX2, CD44, LGR5, NANOG, and ALDH1) in GMDS-AS1-silenced HCT116 (above) and SW620 (below) cells compared with control cells. **D.** Sphere-forming assay with control and stable GMDS-AS1-expressing RKO cells. Left, representative images; right, quantification of the spheres. Scale bar, 200 μm. **E.** Relative expression levels of CSC-related genes in control and stable GMDS-AS1-expressing RKO cells. **F**. *In vitro* ELDA assay of control and GMDS-AS1 KD HCT116 cells. Left, quantification of the sphere-initiating cell frequency. Right, graphic statistics; **G.** Representative tumor image of *In vivo* ELDA assay formed by 3 doses of control and GMDS-AS1 KD HCT116 cells. **H.** Quantification of the tumor-initiating frequency in **G**. **I.** Tumor volume and tumor weight of the mice shown in **G** were measured. **J.** CD133^+^CD44^+^ and CD166^+^ subpopulations were detected in control and GMDS-AS1 KD HCT116 cells by FACS and plotted. The percentage of positive cells was quantified. **A-E, I, J:** Values are expressed as the means ± SEM, n = 3. ***P < 0.001, **P < 0.01, and *P < 0.05 by two-tailed Student’s t test.

**Figure S5. GMDS-AS1 upregulates the expression of STAT3 target genes**

**A, B.** Relative mRNA levels of STAT3 target genes in HCT116 cells (A) and SW620 cells (B) stably transduced with control or GMDS-AS1 short hairpin RNA (shRNA) were measured by qPCR. 18S was the endogenous control. **C.** Relative mRNA expression levels of STAT3 target genes in RKO cells stably expressing control or GMDS-AS1 plasmids were measured by qPCR. 18S was the endogenous control. **D.** Luciferase activity assays of the STAT3 signaling pathway in RKO cells stably expressing control or GMDS-AS1 ASO. **E.** Luciferase activity assays of the Wnt signaling pathway (TOP/FOP flash activity measurement) in RKO cells stably expressing control or GMDS-AS1 ASO. **F.** Luciferase activity assays of the Wnt signaling pathway (TOP/FOP flash activity measurement) in HCT116 cells stably expressing control or GMDS-AS1 shRNAs. **G.** Luciferase activity assays of the Wnt signaling pathway (TOP/FOP flash activity measurement) in HCT116 and RKO cells stably expressing control or GMDS-AS1 plasmids. **H.** Relative mRNA levels of GMDS-AS1 in HCT116 and RKO cells stably expressing control or GMDS-AS1 plasmids with or without Wnt3a. **I.** Relative mRNA levels of Wnt/β-catenin target genes in HCT116 and RKO cells stably expressing control or GMDS-AS1 shRNAs. **J, K** Relative mRNA levels of STAT3 target and apoptosis related genes in xenografted tumors from control or GMDS-AS1 KD HCT116 cells (J) and control or GMDS-AS1 OE RKO cells (K). RNA expression was measured by qPCR. 18S was the endogenous control. **L, M** Relative mRNA levels of Wnt target and CSC/EMT-related genes in xenografted tumors from control or GMDS-AS1 KD HCT116 cells (L) and control or GMDS-AS1 OE RKO cells (M). RNA expression were measured by qPCR. 18S was the endogenous control. **A-I.** Values are expressed as the means ± SEM, n=3. ***P < 0.001, **P < 0.01, and *P < 0.05 by two-tailed Student’s t test.

**Figure S6. GMDS-AS1 does not directly bind to the STAT3 protein or regulate the transcription of STAT3 mRNA**

**A.** RNA immunoprecipitation (RIP) experiments confirmed that the long noncoding RNA (lncRNA) GMDS-AS1 and the STAT3 protein do not directly bind. **B.** Relative mRNA expression levels of the *STAT3* gene in spheres consisting of RKO cells stably expressing control or GMDS-AS1 plasmids were measured by qPCR. 18S was the endogenous control. **C.** Relative pre-mRNA expression levels of the *STAT3* gene in HCT116 cells stably transduced with control or GMDS-AS1 shRNA were measured by qPCR. Two pairs of qPCR primers were designed to ensure the reliability of the results. 18S was the endogenous control. **D.** GMDS-AS1 and *STAT3* RNA expression levels in 23 pairs of CRC tissues and adjacent normal tissues were detected by qPCR. **E.** Correlation analysis of GMDS-AS1 and STAT3 mRNA expression levels in The Cancer Genome Atlas (TCGA) colon adenocarcinoma (COAD) dataset cohort. The expression level was normalized to that of the GAPDH gene. The figure was generated with the Gene Expression Profiling Interactive Analysis 2 (GEPIA2) tool. **F.** Deletion mapping was performed to identify the STAT3 binding domain in HuR in HCT116 cells. Plasmids expressing a Flag-tag, Flag-tagged full-length HuR or Flag-tagged truncated HuR were transfected into HCT116 cells, and RIP experiments were performed using antibodies against Flag. qPCR was performed to detect the STAT3 enrichment levels. **G.** HuR binding sites on GMDS-AS1 were predicted by RBPmap (<http://rbpmap.technion.ac.il/index.html>). **H.** Replicates of Figure 3H. **I, J** Replicates of Figure 6A. **A-C**: Values are expressed as the means ± SEM, n=3. ***P < 0.001, **P < 0.01, and *P < 0.05 by two-tailed Student’s t test.

**Figure S7. GMDS-AS1 does not affect HuR mRNA expression**

**A.** Relative mRNA levels of *HuR (ELAVL1)* in HCT116 cells stably transduced with control or GMDS-AS1 short hairpin RNA (shRNA) were measured by qPCR. **B.** Relative mRNA expression levels of *HuR (ELAVL1)* in control and stable GMDS-AS1-expressing RKO cells were measured by qPCR. **C.** Relative mRNA expression levels of *HuR (ELAVL1)* in HCT116 cells stably transduced with control or HuR shRNA were measured by qPCR. **D.** Relative mRNA expression levels of STAT3 target genes in HCT116 cells stably transduced with control or HuR shRNA were measured by qPCR. **E.** HCT116 cells stably transduced with control or HuR shRNA were treated with cycloheximide (CHX; 50 μg/mL) for the indicated times, and the protein expression level of HuR was detected by immunoblotting (left). Densitometric analysis curve of HuR protein levels (right). β-actin was the endogenous control. **F.** Relative pre-mRNA expression levels of HuR (*ELAVL1*) in HCT116 cells stably transduced with control or GMDS-AS1 shRNA were measured by qPCR. **G.** Correlation analysis of HuR and STAT3 mRNA levels in The Cancer Genome Atlas (TCGA) colon adenocarcinoma (COAD) dataset cohort. The expression level was normalized to that of the GAPDH gene. The figure was generated with the Gene Expression Profiling Interactive Analysis 2 (GEPIA2) tool. *** P < 0.001, ** P < 0.01, and *P < 0.05 by two-tailed Student’s t test.
