# Supplementary figures and images for: LncGMDS-AS1 promotes the tumorigenesis of colorectal cancer through HuR-STAT3/Wnt axis
Source: Cell Death Dis. 2023 Feb 27;14(2):165. doi: 10.1038/s41419-023-05700-8 (PMC9970971; doi:10.1038/s41419-023-05700-8)

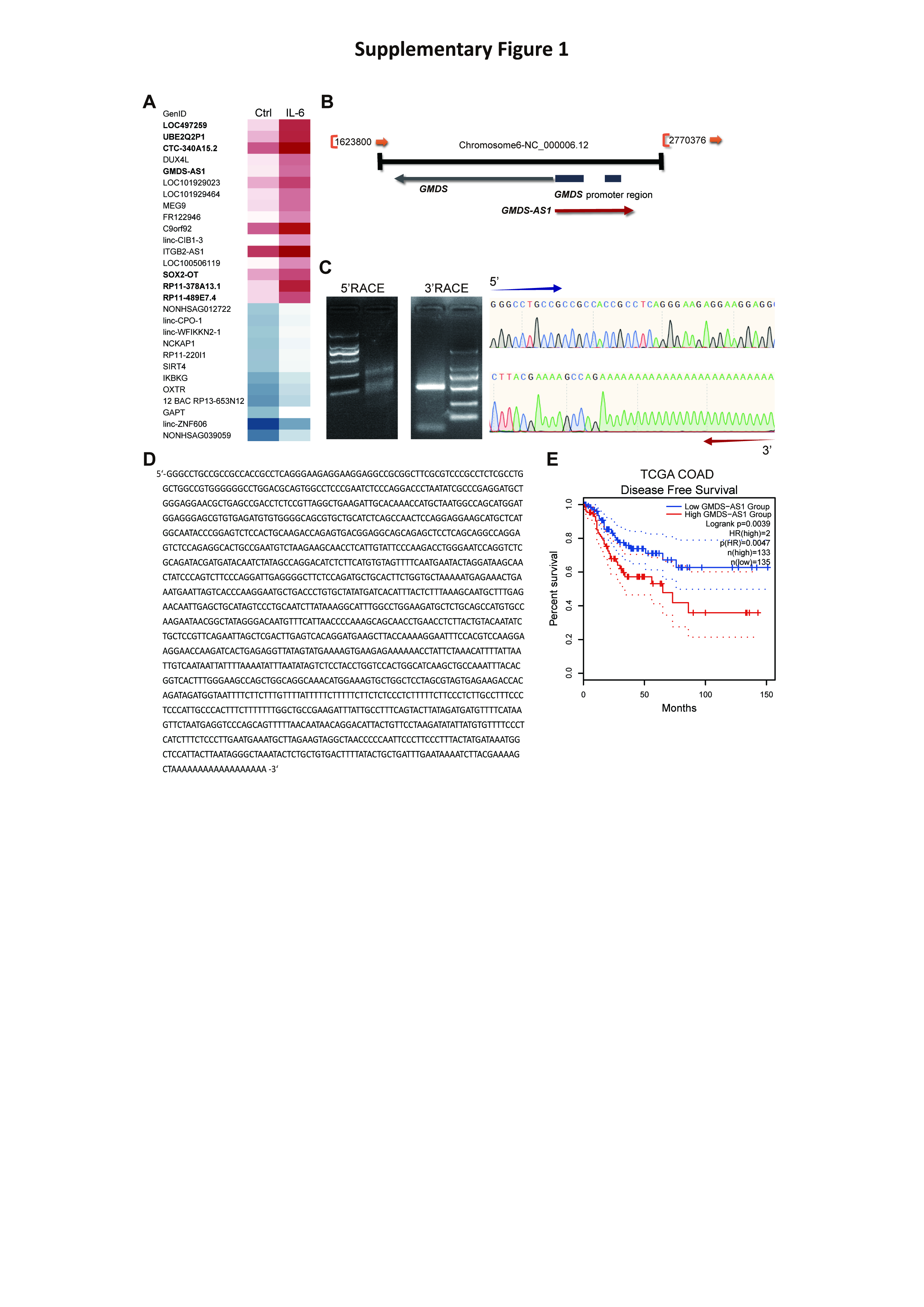

Supplement: Supplementary file 2 — Figure S1 [file 41419_2023_5700_MOESM2_ESM.tif]

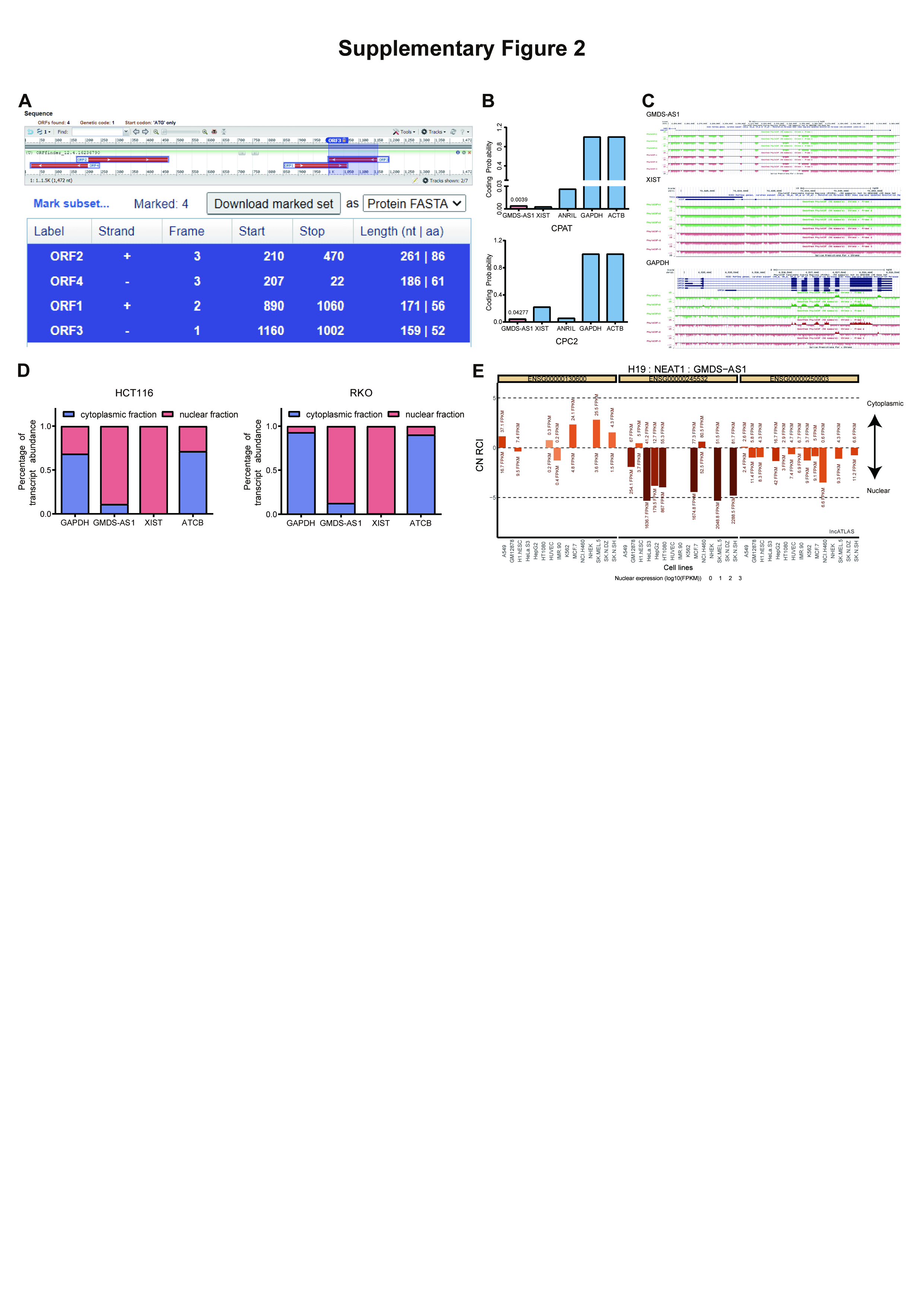

Supplement: Supplementary file 3 — Figure S2 [file 41419_2023_5700_MOESM3_ESM.tif]

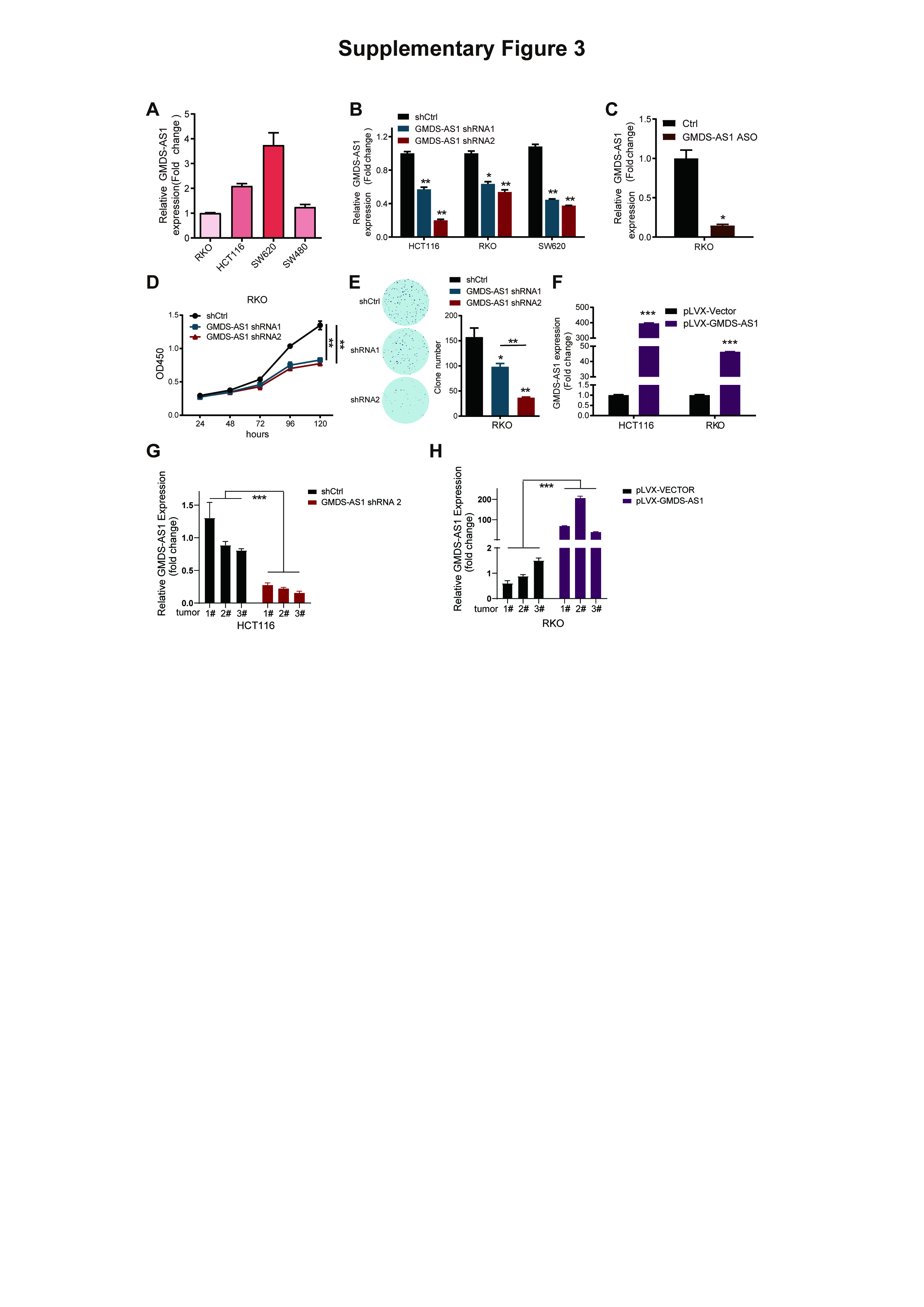

Supplement: Supplementary file 4 — Figure S3 [file 41419_2023_5700_MOESM4_ESM.tif]

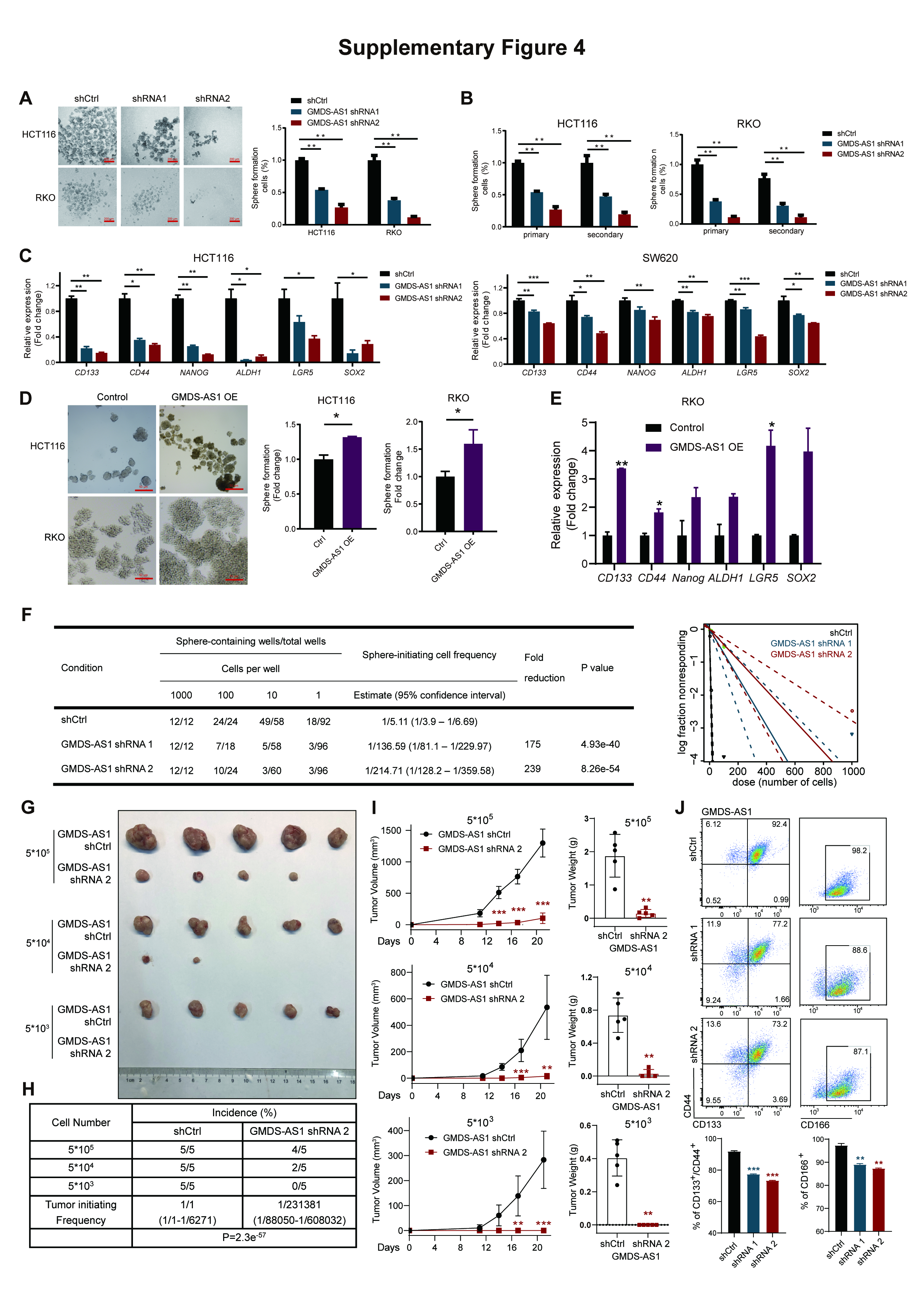

Supplement: Supplementary file 5 — Figure S4 [file 41419_2023_5700_MOESM5_ESM.tif]

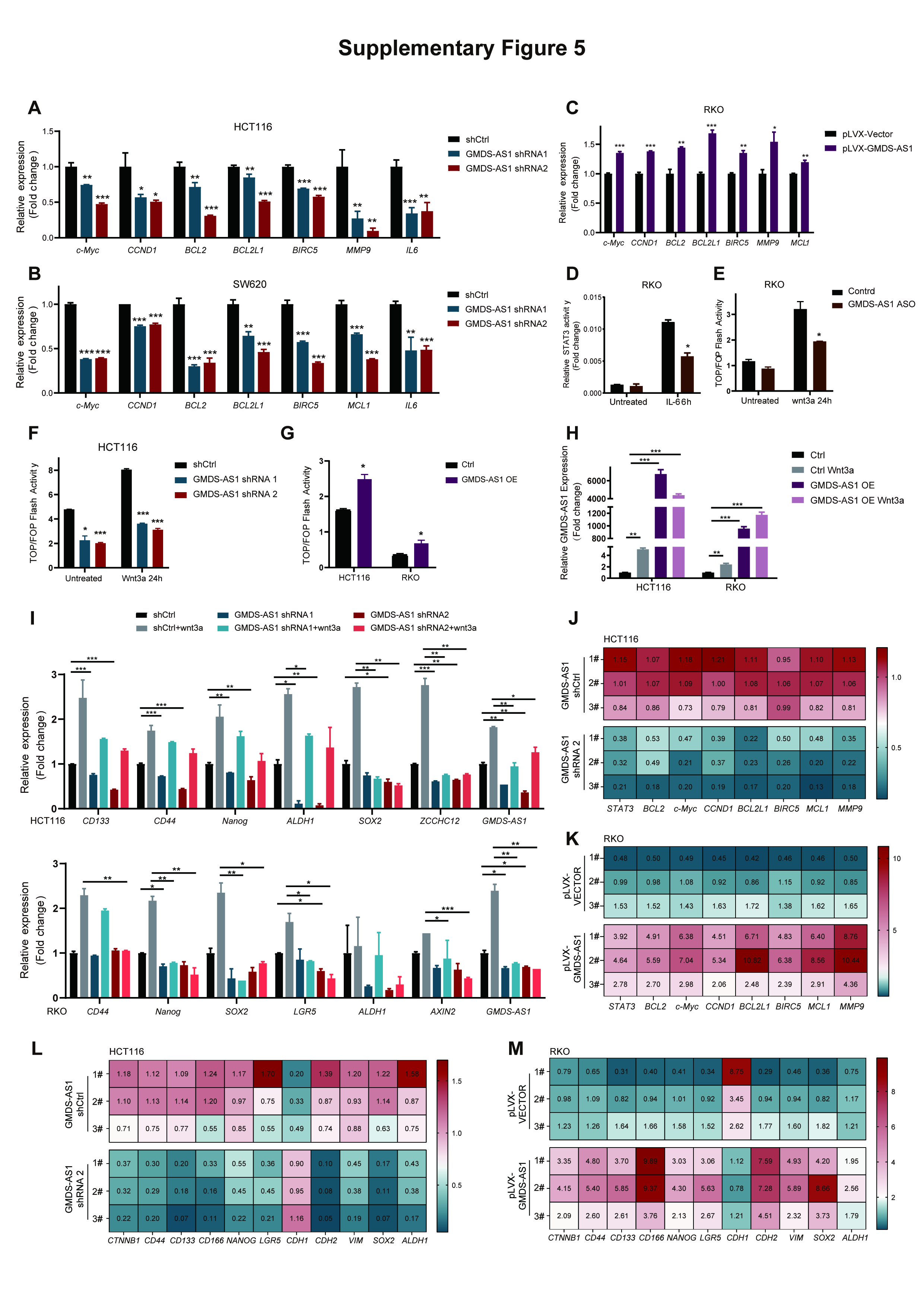

Supplement: Supplementary file 6 — Figure S5 [file 41419_2023_5700_MOESM6_ESM.tif]

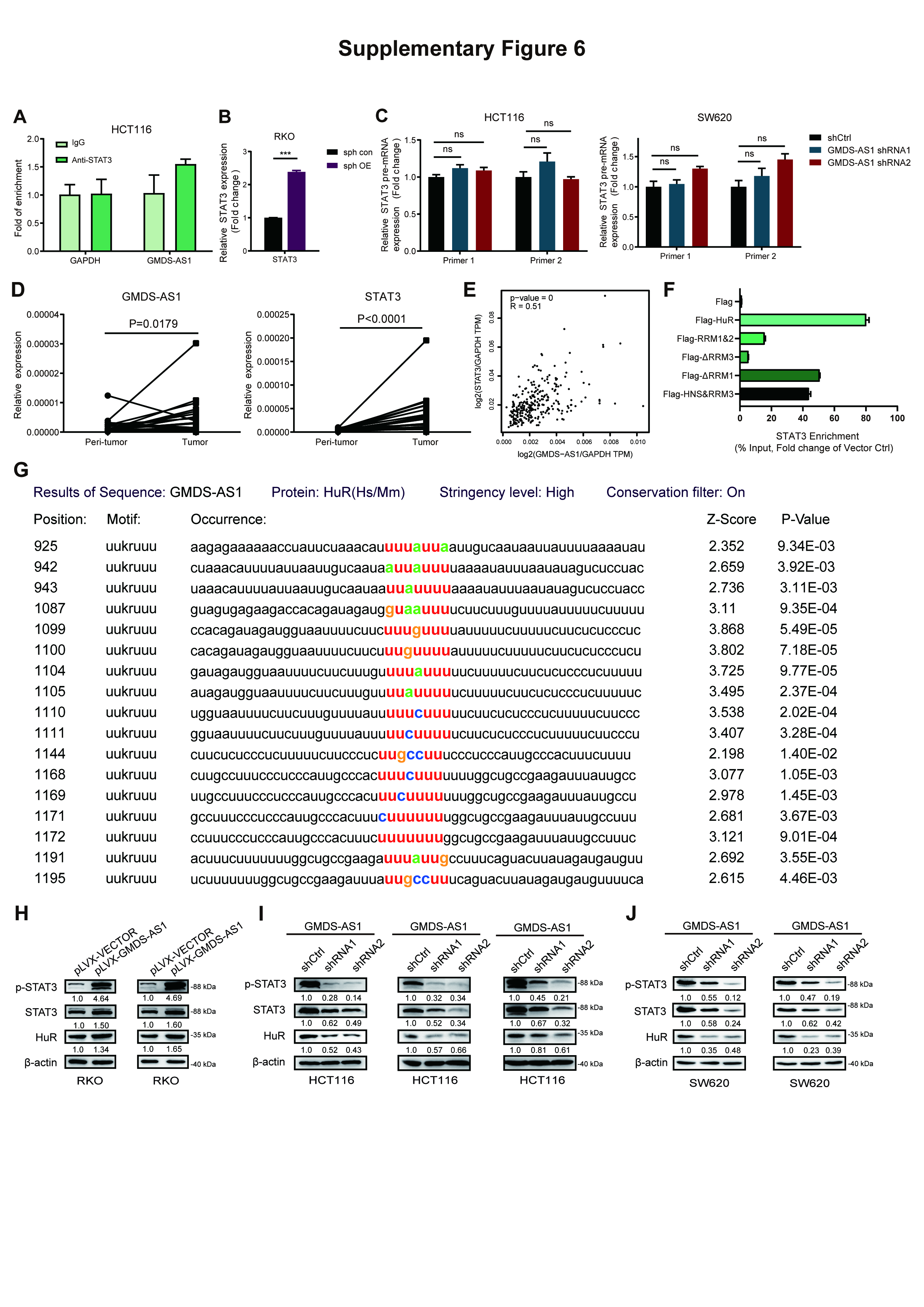

Supplement: Supplementary file 7 — Figure S6 [file 41419_2023_5700_MOESM7_ESM.tif]

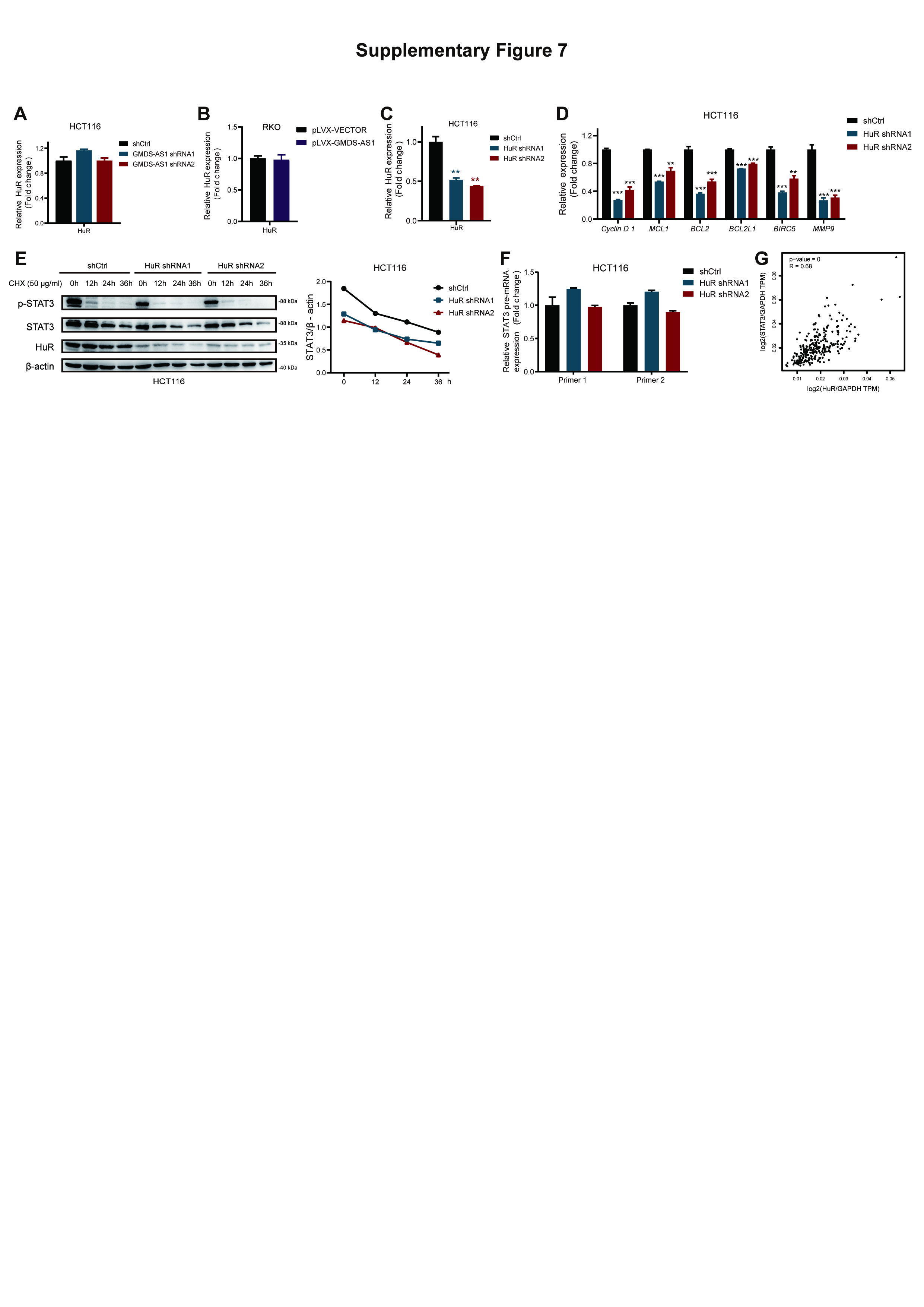

Supplement: Supplementary file 8 — Figure S7 [file 41419_2023_5700_MOESM8_ESM.tif]

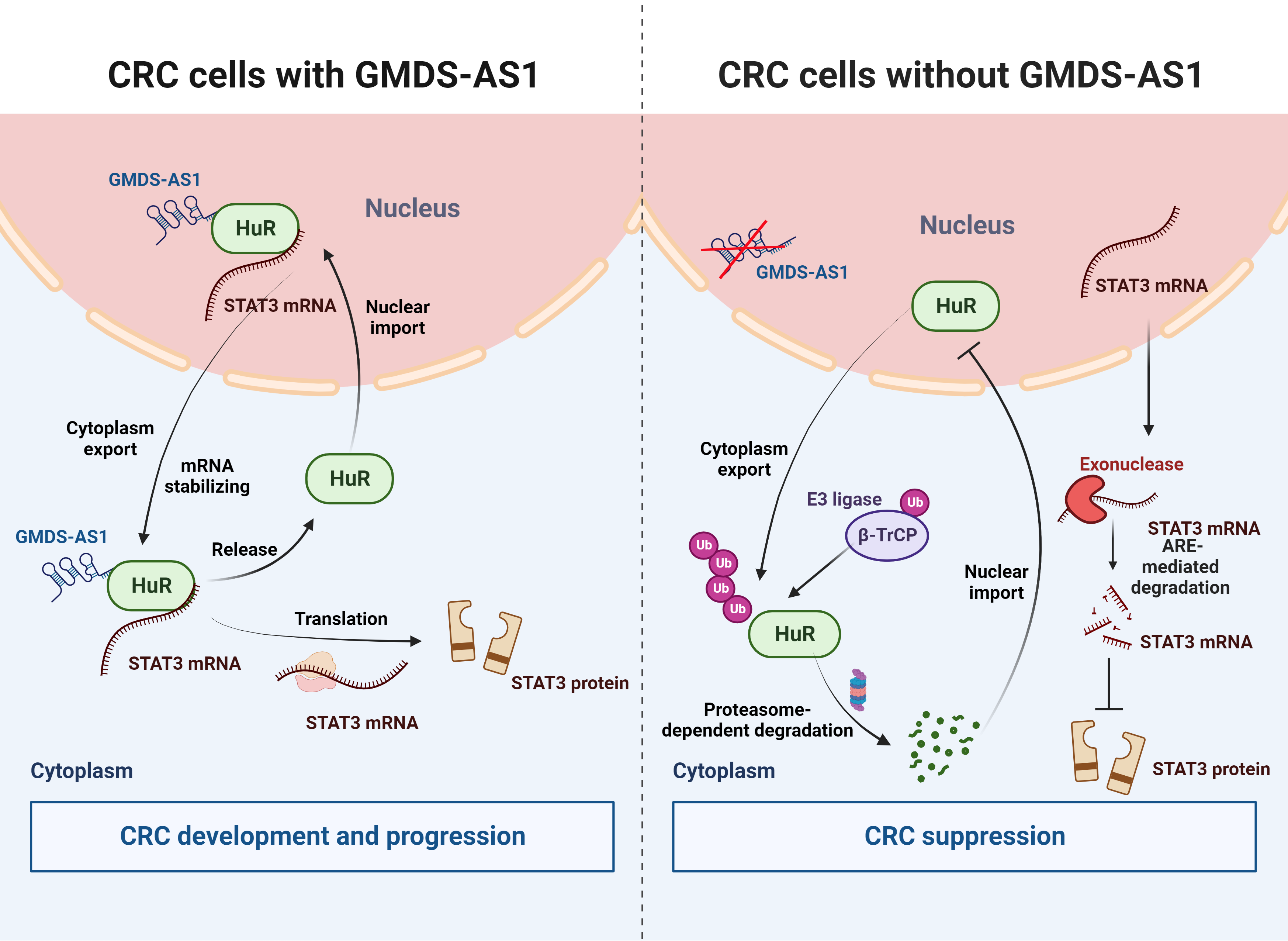

Supplement: Supplementary file 15 — GMDS-AS1-HuR-STAT3 axis [file 41419_2023_5700_MOESM15_ESM.png]
