# Supplementary material for: LncGMDS-AS1 promotes the tumorigenesis of colorectal cancer through HuR-STAT3/Wnt axis
Source: Cell Death Dis. 2023 Feb 27;14(2):165. doi: 10.1038/s41419-023-05700-8 (PMC9970971; doi:10.1038/s41419-023-05700-8)

Figure 2F

HCT116

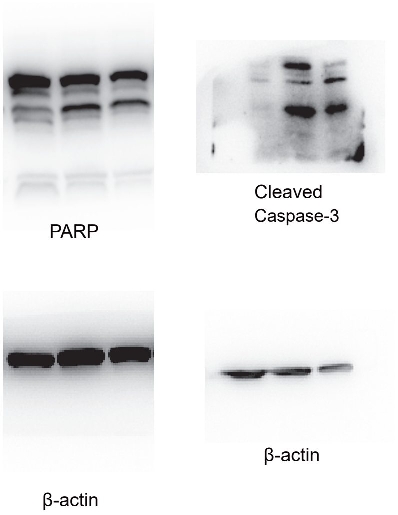

SW620

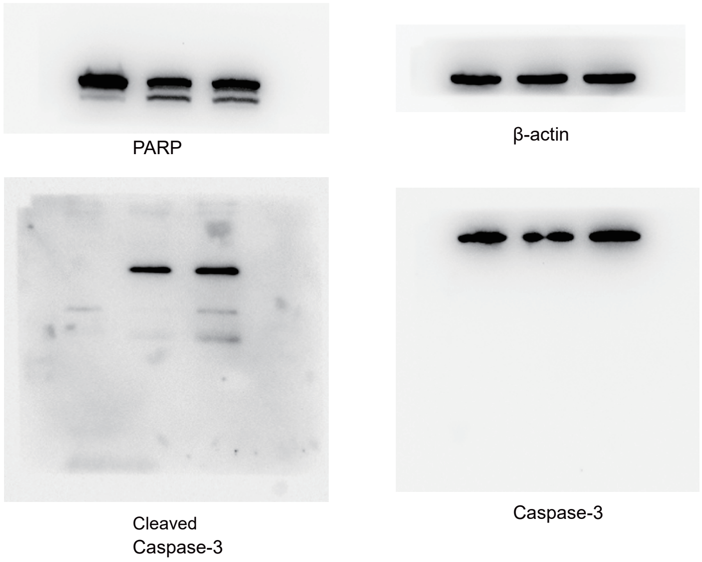

Figure 3F

HCT116

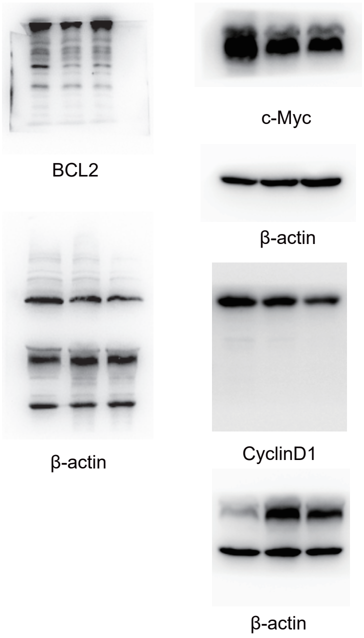

SW620

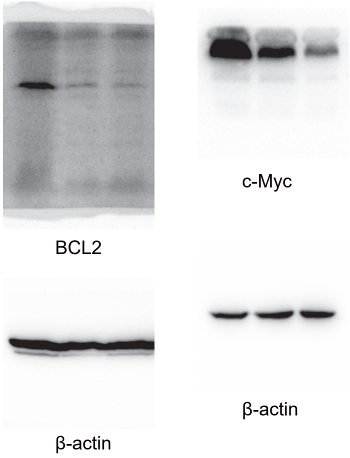

Figure 3G RKO

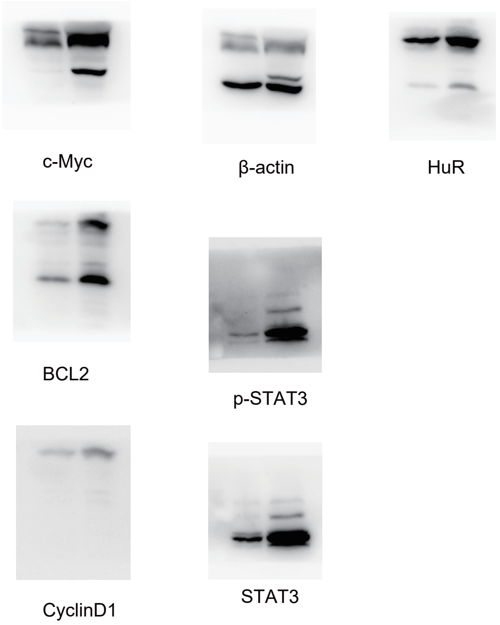

Figure 4A

RKO

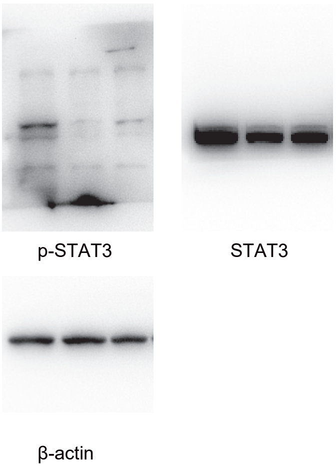

HCT116

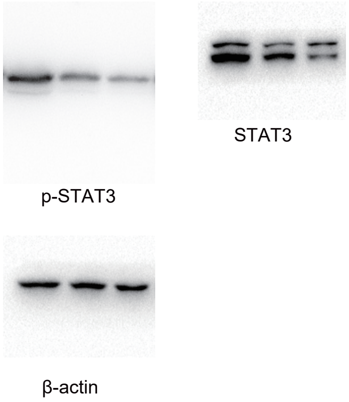

SW620

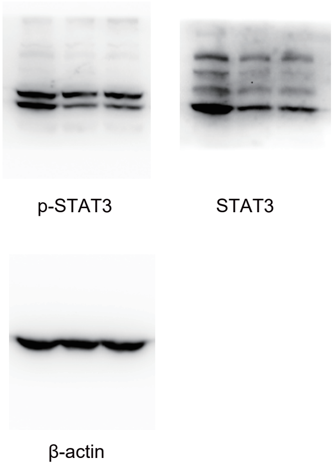

Figure 4B RKO

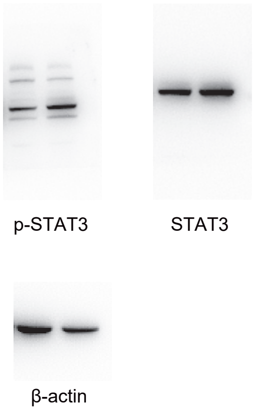

Figure 4C

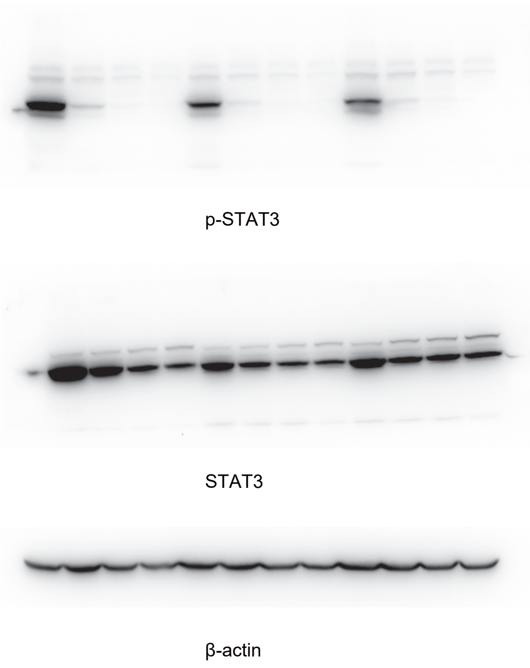

Figure 5A

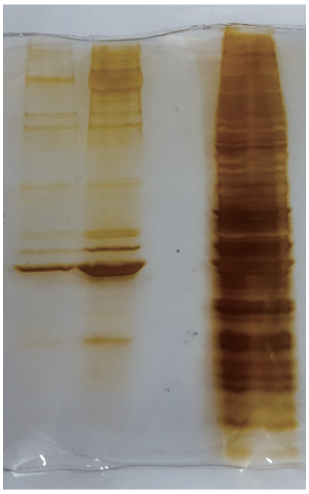

Figure 5B

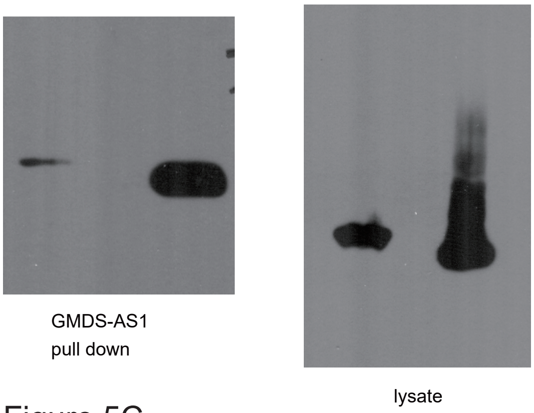

Figure 5C

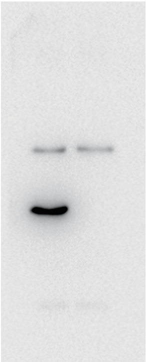

Figure 5E

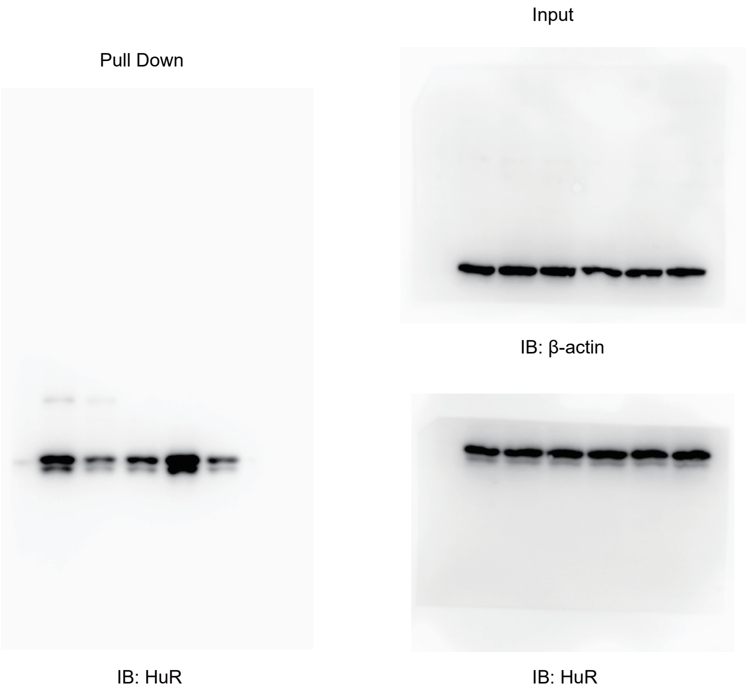

Figure 6A

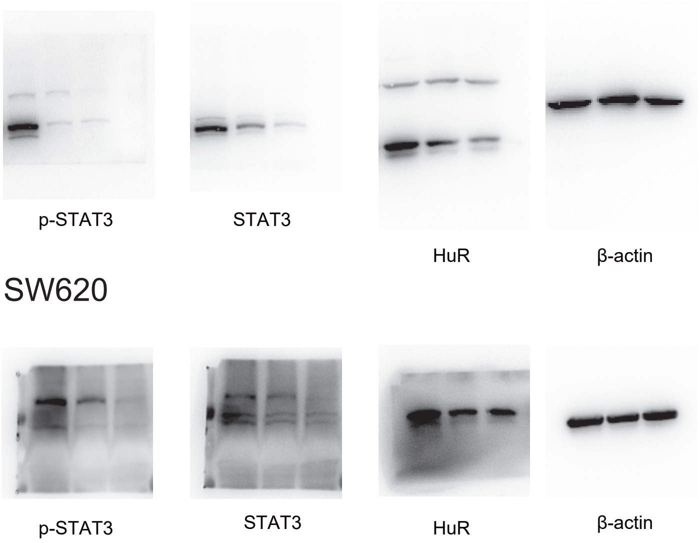

Figure 6B

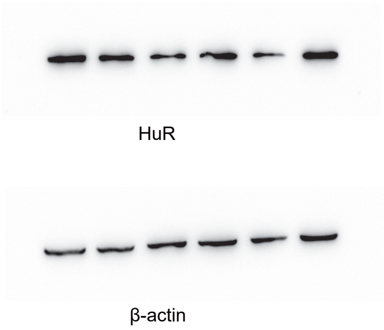

Figure 6C

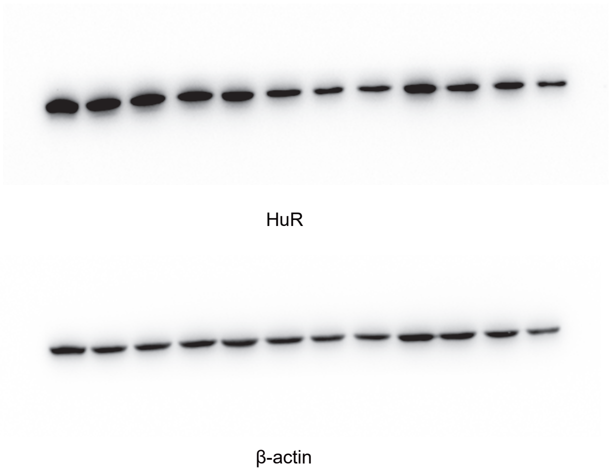

Figure 6D

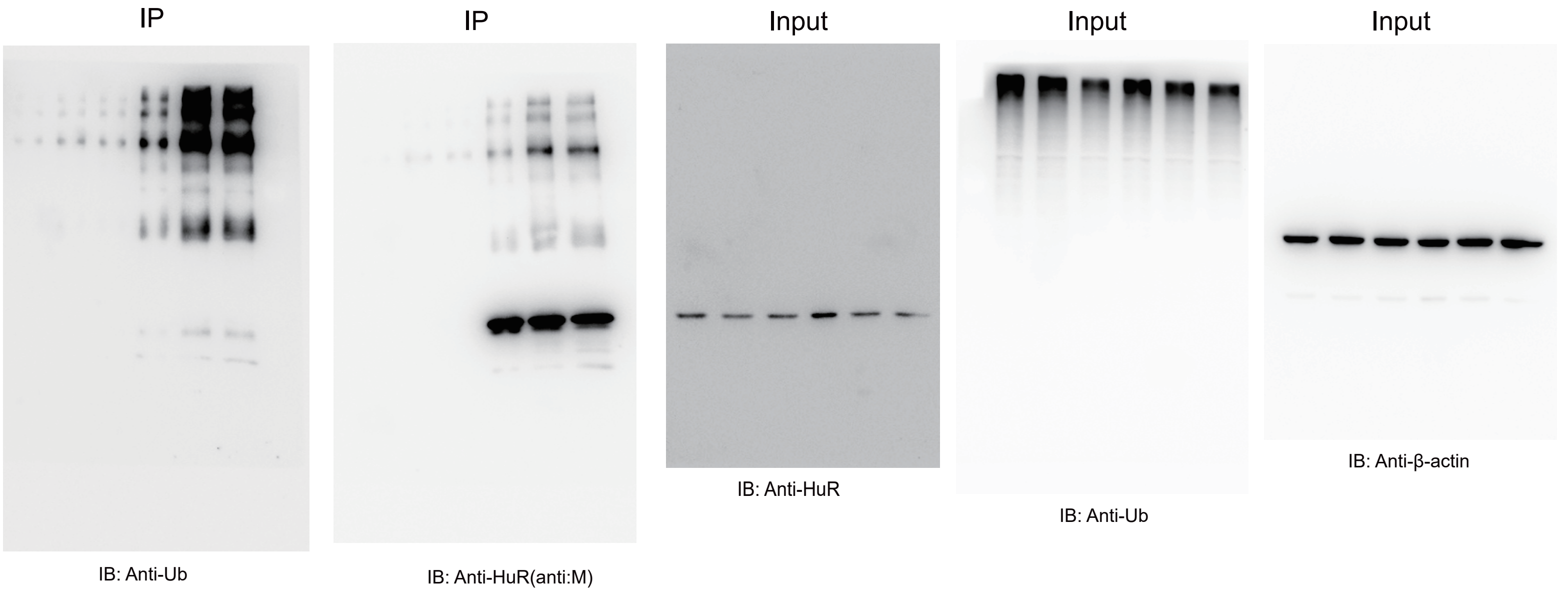

Figure 6E

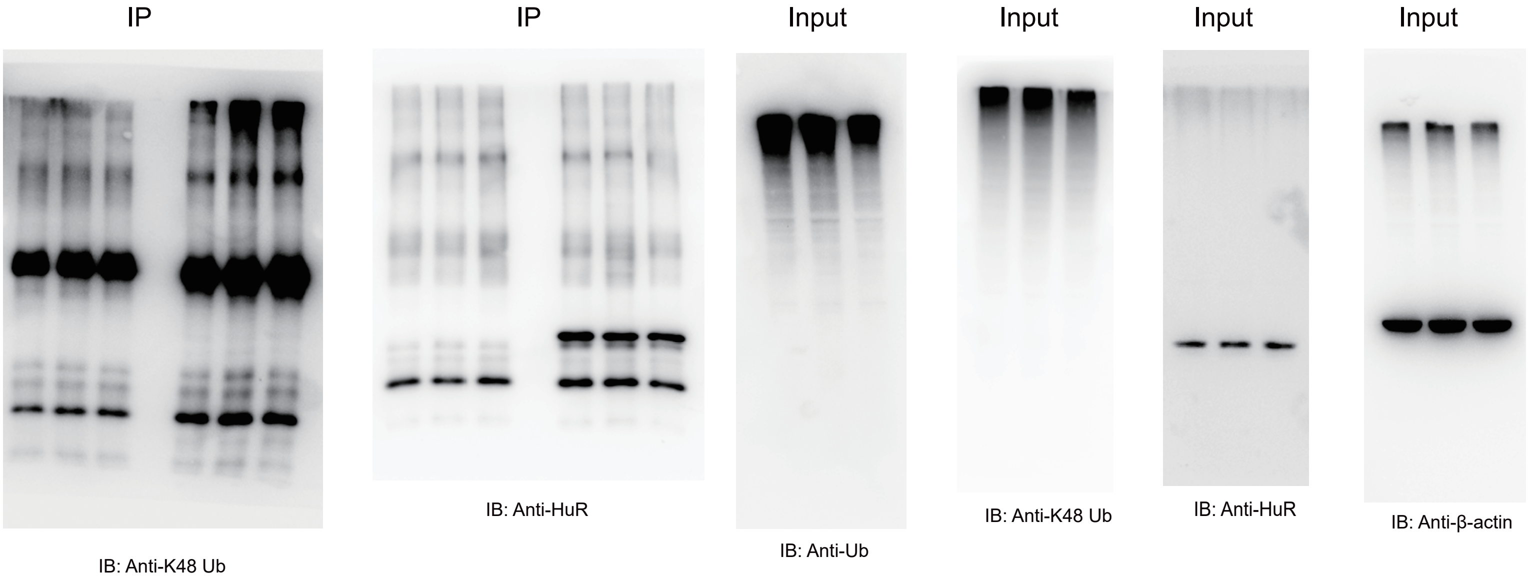

Figure 6F

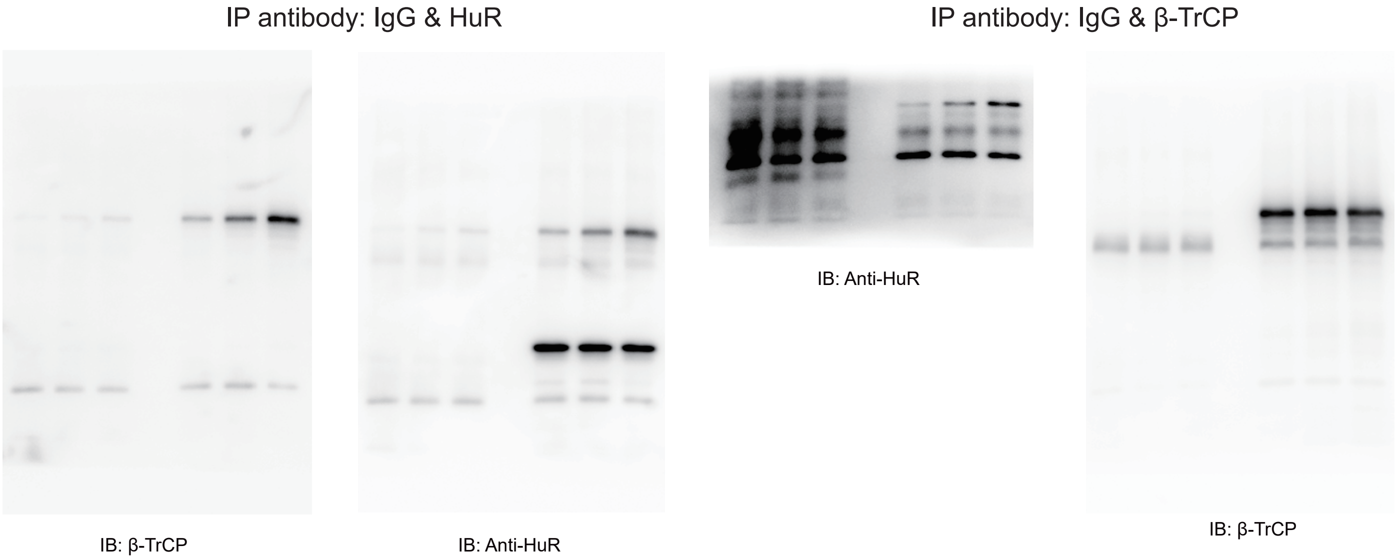

Figure 6F

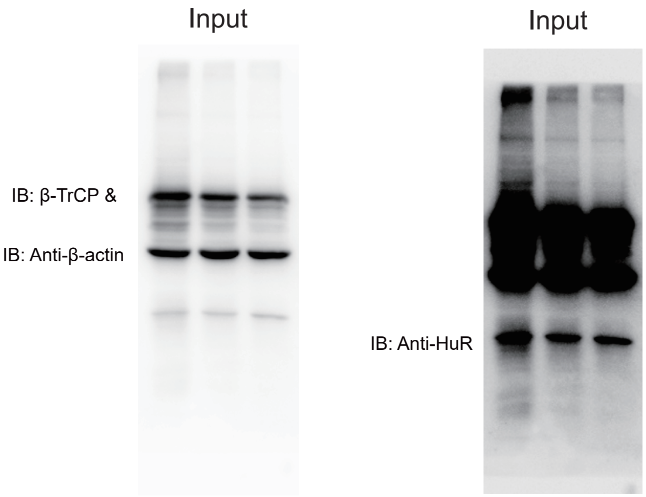

Figure 7A

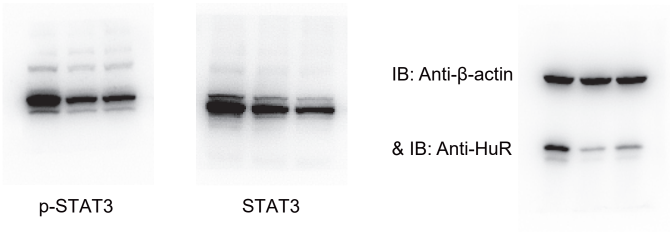

Figure 7D

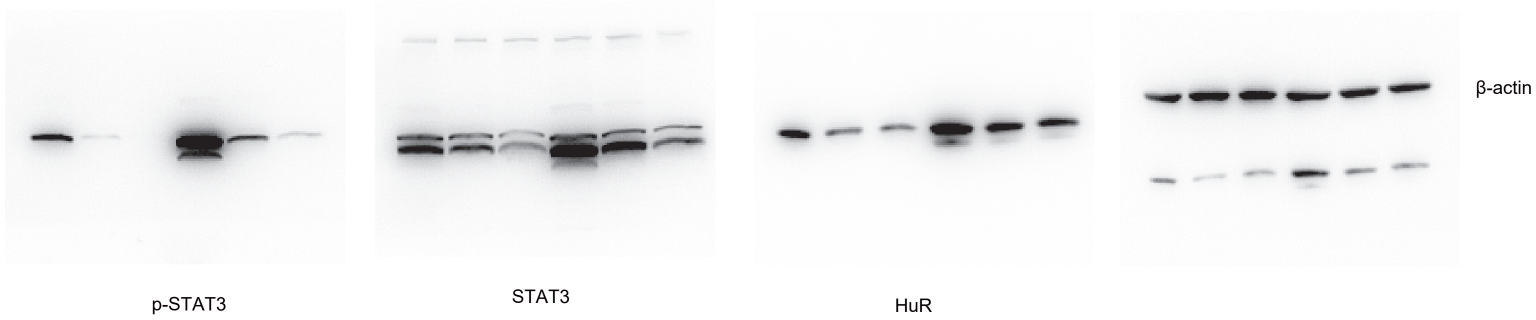

Figure S6H

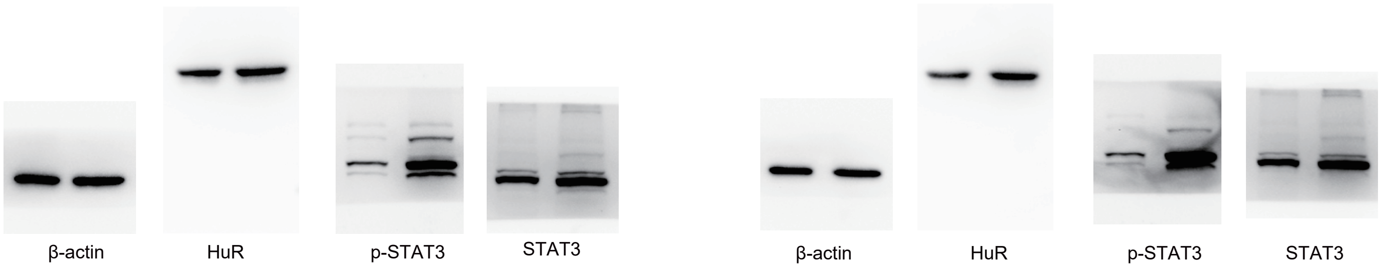

Figure S6I

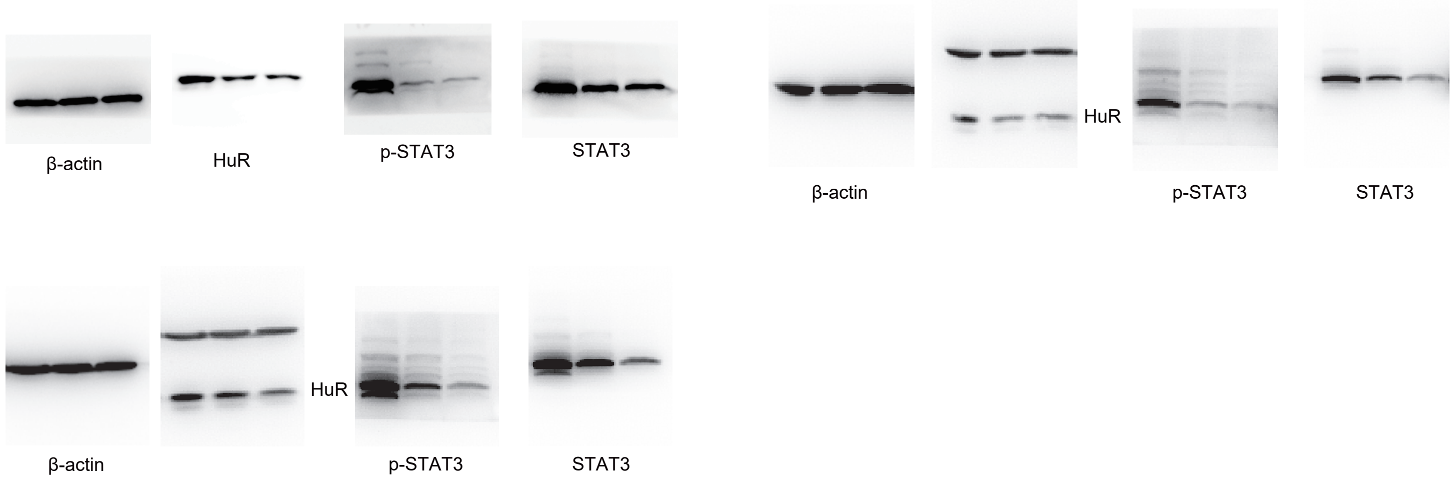

Figure S6J

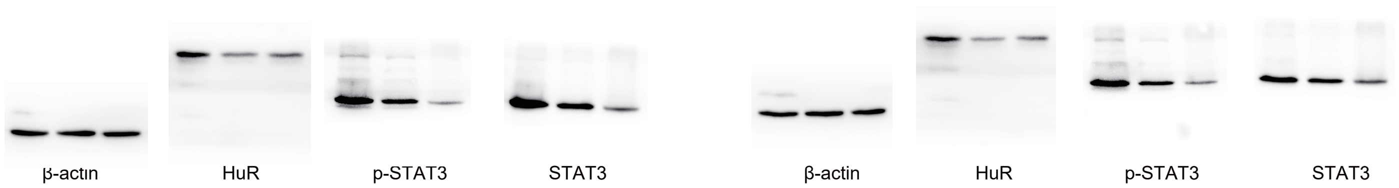

β-actin HuR p-STAT3 STAT3 β-actin HuR p-STAT3 STAT3

Figure S7E

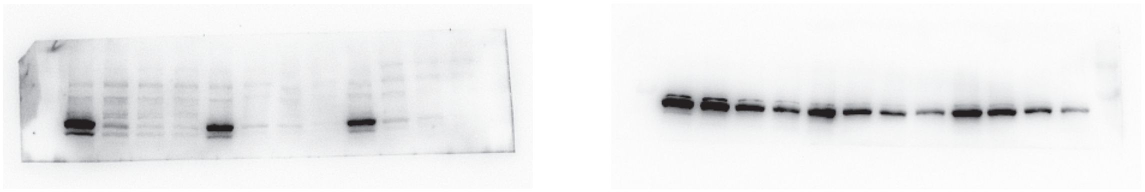

p-STAT3 STAT3

HuR

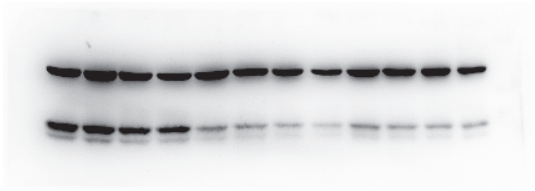

β-actin

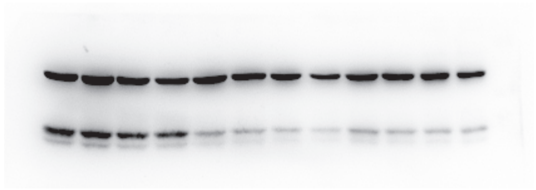

Supplement: Supplementary file 16 — Original Western Blot Image [file 41419_2023_5700_MOESM16_ESM.pdf]
